# Independent Evolution of Sex Chromosomes and Male Pregnancy–Related Genes in Two Seahorse Species

**DOI:** 10.1093/molbev/msac279

**Published:** 2022-12-29

**Authors:** Xin Long, Deborah Charlesworth, Jianfei Qi, Ruiqiong Wu, Meiling Chen, Zongji Wang, Luohao Xu, Honggao Fu, Xueping Zhang, Xinxin Chen, Libin He, Leyun Zheng, Zhen Huang, Qi Zhou

**Affiliations:** MOE Laboratory of Biosystems Homeostasis and Protection and Zhejiang Provincial Key Laboratory for Cancer Molecular Cell Biology, Life Sciences Institute, Zhejiang University, Hangzhou 310058, China; Research Center for Intelligent Computing Platforms, Zhejiang Lab, Hangzhou 311100, China; Institute of Evolutionary Biology, School of Biological Sciences, University of Edinburgh, West Mains Road, Edinburgh EH9 3LF, UK; Department of Aquaculture, Fisheries Research Institute of Fujian, Xiamen 361013, China; Fujian Key Laboratory of Developmental and Neural Biology & Southern Center for Biomedical Research, College of Life Sciences, Fujian Normal University, Fuzhou, Fujian, China; Fujian Key Laboratory of Developmental and Neural Biology & Southern Center for Biomedical Research, College of Life Sciences, Fujian Normal University, Fuzhou, Fujian, China; MOE Laboratory of Biosystems Homeostasis and Protection and Zhejiang Provincial Key Laboratory for Cancer Molecular Cell Biology, Life Sciences Institute, Zhejiang University, Hangzhou 310058, China; MOE Key Laboratory of Freshwater Fish Reproduction and Development, Key Laboratory of Aquatic Science of Chongqing, School of Life Sciences, Southwest University, Chongqing 400715, China; Fujian Key Laboratory of Developmental and Neural Biology & Southern Center for Biomedical Research, College of Life Sciences, Fujian Normal University, Fuzhou, Fujian, China; Fujian Key Laboratory of Developmental and Neural Biology & Southern Center for Biomedical Research, College of Life Sciences, Fujian Normal University, Fuzhou, Fujian, China; Department of Aquaculture, Fisheries Research Institute of Fujian, Xiamen 361013, China; Department of Aquaculture, Fisheries Research Institute of Fujian, Xiamen 361013, China; Department of Aquaculture, Fisheries Research Institute of Fujian, Xiamen 361013, China; Fujian Key Laboratory of Developmental and Neural Biology & Southern Center for Biomedical Research, College of Life Sciences, Fujian Normal University, Fuzhou, Fujian, China; Fujian-Macao Science and Technology Cooperation Base of Traditional Chinese Medicine-Oriented Chronic Disease Prevention and Treatment, Innovation and Transformation Center, Fujian University of Traditional Chinese Medicine, Fuzhou 350108, China; MOE Laboratory of Biosystems Homeostasis and Protection and Zhejiang Provincial Key Laboratory for Cancer Molecular Cell Biology, Life Sciences Institute, Zhejiang University, Hangzhou 310058, China; MOE Key Laboratory of Freshwater Fish Reproduction and Development, Key Laboratory of Aquatic Science of Chongqing, School of Life Sciences, Southwest University, Chongqing 400715, China; Center for Reproductive Medicine, The Second Affiliated Hospital, School of Medicine, Zhejiang University, Hangzhou 310052, China; Evolutionary & Organismal Biology Research Center, School of Medicine, Zhejiang University, Hangzhou 310058, China

**Keywords:** seahorse, sex chromosome, genome evolution

## Abstract

Unlike birds and mammals, many teleosts have homomorphic sex chromosomes, and changes in the chromosome carrying the sex-determining locus, termed “turnovers”, are common. Recent turnovers allow studies of several interesting questions. One question is whether the new sex-determining regions evolve to become completely non-recombining, and if so, how and why. Another is whether (as predicted) evolutionary changes that benefit one sex accumulate in the newly sex-linked region. To study these questions, we analyzed the genome sequences of two seahorse species of the Syngnathidae, a fish group in which many species evolved a unique structure, the male brood pouch. We find that both seahorse species have XY sex chromosome systems, but their sex chromosome pairs are not homologs, implying that at least one turnover event has occurred. The Y-linked regions occupy 63.9% and 95.1% of the entire sex chromosome of the two species and do not exhibit extensive sequence divergence with their X-linked homologs. We find evidence for occasional recombination between the extant sex chromosomes that may account for their homomorphism. We argue that these Y-linked regions did not evolve by recombination suppression after the turnover, but by the ancestral nature of the low crossover rates in these chromosome regions. With such an ancestral crossover landscape, a turnover can instantly create an extensive Y-linked region. Finally, we test for adaptive evolution of male pouch–related genes after they became Y-linked in the seahorse.

## Introduction

Studies of young sex-linked regions are important for understanding the processes involved in the initial evolution of sex chromosomes, including how their lack of recombination arose, and the time-course of subsequent adaptive and degenerative changes (reviewed by Bachtrog, et al. 2014). The discovery that sex-determining regions (SDRs) have evolved independently in different fish species (reviewed by Pan, et al. 2016) offers opportunities for such studies. A major idea to explain the lack of recombination between sex chromosomes is the sexually antagonistic (SA) polymorphism hypothesis, involving selection generated by conflicts or fitness trade-offs between alleles’ effects in the two sexes (Rice 1987). Such selection can establish a two-locus polymorphism with alleles at the locus under SA selection associated with alleles at the sex-determining locus, which generates selection for closer linkage between the two genes (Bull 1983). This process of expanding the non-recombining region can be repeated, causing successive recombination suppression events, at different evolutionary times, each with a distinctive level of sequence divergence between Y and X or W and Z sex-linked gene pairs. Such so-called “evolutionary strata” have been detected in mammalian XY chromosome pairs (Lahn and Page 1999; Bellott, et al. 2014; Cortez, et al. 2014). It was proposed that these recombination suppression events are caused by inversions (Lahn and Page 1999), and there is evidence supporting this for at least one human stratum (Lemaitre, et al. 2009), and in papaya (Wang, et al. 2012), and perhaps in the threespine stickleback (Peichel, et al. 2020). Recombination suppression does not require an inversion, and can also evolve via recombination modifiers that control crossovers in specific genome locations. Other species with female heterogamety, including birds, also appear to have evolutionary strata (Zhou, et al. 2014).

In contrast to the ancient mammalian and bird sex chromosomes, non-homology of the sex chromosomes of closely related species has been documented in several teleosts, amphibians, and plants, indicating that changes, or “turnover events” have occurred (reviewed by Vicoso 2019), sometimes even between different populations of the same species (e.g., a frog [Miura, et al. 2012]). Turnovers might also involve SA polymorphisms, as such a polymorphism can favor the appearance of a new sex-determining gene in a closely linked genomic region or can become established near a sex-determining gene that has appeared in a new genome region for some other reason (van Doorn and Kirkpatrick 2007).

Turnover events can offer opportunities to test the SA polymorphism hypothesis. This hypothesis is extremely difficult to test (see, for example Olito, et al. 2022) because it is challenging to document a balanced polymorphism that is being maintained is challenging; and the additional requirement for evidence that SA selection is involved makes the task even harder, though population genomic approaches are starting to be developed (Qiu, et al. 2013; Dagilis, et al. 2022).

While old-established turnovers can be investigated to test whether new completely sex-linked strata have subsequently evolved, recent turnover events can reveal alternative origins of large sex-linked genome regions. Because SA polymorphisms are expected to be maintained mainly at loci very closely linked to the sex-determining locus (Jordan and Charlesworth 2012), they are unlikely to lead to the evolution of large sex-linked genome regions unless a large inversion happens to suppress recombination across the region that includes the SA and sex-determining loci, and becomes fixed in the population; invoking recurrent such events in multiple organisms seems implausible (Olito, et al. 2022). Therefore, alternative possibilities should be considered if an extensive sex-linked region is found after a recent turnover.

A complementary approach is to test whether the genome region linked to a new male-determining locus starts accumulating SA mutations. Under male heterogamety, for example, mutations can spread if they benefit aspects of male fitness enough to outweigh any deleterious effects in females. Even if the restrictive conditions are not satisfied for such mutations to establish polymorphisms (Jordan and Charlesworth 2012) and create a selection for linkage with the male-determining gene, their accumulation or fixation in the population (replacing both the ancestral Y- and X-linked alleles) can potentially be detected without needing to detect balancing selection. The Syngnathidae is suitable for such studies because, uniquely among animals, they have evolved the male brood pouch, in which males fertilize the eggs and nurture the offspring until hatching (Whittington, et al. 2015).

We here study several species of Syngnathidae, a teleost group that includes seahorses, seadragons, and pipefish (Stiller, et al. 2022). The syngnathids’ male pregnancy reproductive behavior is unique among animals, and its evolution appears to have involved modifications of the immune gene repertoire, including the loss or rapid sequence evolution of several major histocompatibility complex (MHC) genes, which may reflect trade-offs between immunological tolerance and embryo rejection (Roth, et al. 2020). There is evidence that male Gulf pipefish actively abort embryos of less attractive females (Paczolt and Jones 2010). Therefore, this group of teleosts has been frequently used to study the evolution of novel morphological traits, parent–offspring conflicts, sexual conflicts, and sexual selection (Vincent, et al. 1992; Lin, et al. 2016; Li, et al. 2021).

**Fig. 1.**
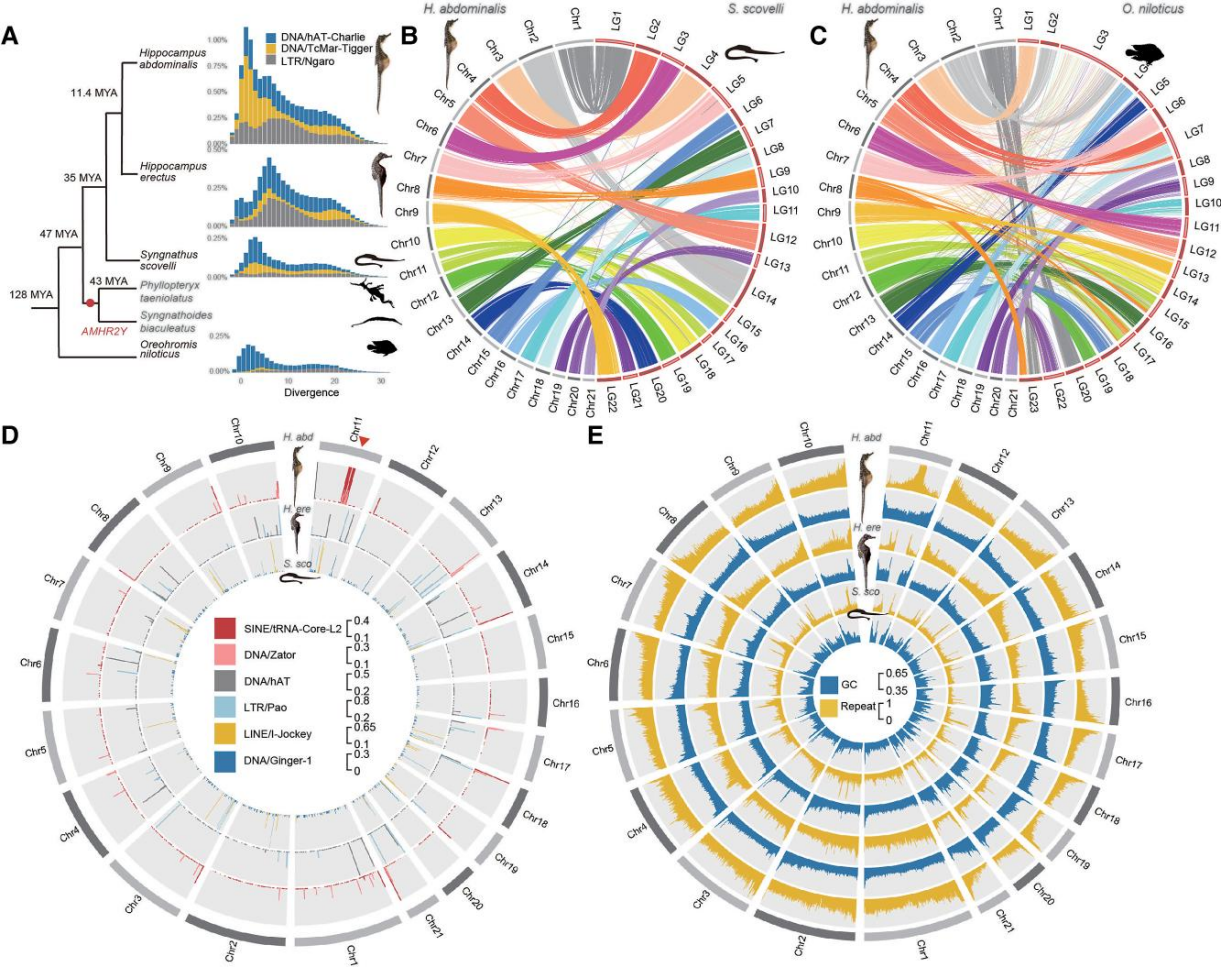
Chromosome evolution and repeat landscapes of seahorse and Gulf pipefish species. (*A*) Phylogenetic tree showing estimated times of appearance (Kumar, et al. 2017) of Syngnathidae species, with the Nile tilapia (*Oreochromis niloticus*) as an outgroup. The dot labels the node corresponding to the time of the previously reported duplication creating the putatively sex-determining gene *AMHR2Y* specific to the two outgroup species to seahorses, *Phyllopteryx taeniolatus* and *Syngnathus biaculeatus* (Supplementary Table S1, see main text). The distributions of sequence divergence from the consensus sequences of the repeat families that have expanded in the two studied seahorse species are also shown. The *Y*-axis represents the genome-wide percentages of repeat families with different divergence values. DNA transposons hAT-Charlie and TcMar-Tigger, as well as LTR Ngaro families have expanded in *Hippocampus abdominalis*, compared with *Hippocampus erectus*, *Syngnathus scovelli*, and *O. niloticus*. The divergence times of these species are labeled on the cladogram at the left of the repeat landscapes. (*B* and *C*) Chromosome synteny between *H. abdominalis* and two other teleosts: Gulf pipefish (*S. scovelli*) and Nile tilapia (*O. niloticus*). Whole genome alignments suggest that most chromosomes of *S. scovelli* and *O. niloticus* have one-to-one homologous relationships with those of *H. abdominalis* except for Chr11. (*D*) Percentage of candidate centromeric repeats in *H. abdominalis, H. erectus,* and *S. scovelli*, shown in the 5 kb sliding window. Candidate centromeric repeats in different species are shown in different tracks. A triangle indicates the putative fusion site of Chr11 in *H. abdominalis*, which corresponds to two homologous chromosomes in the other two teleosts. (*E*) GC and overall repeat content *of H. abdominalis, H. erectus,* and *S. scovelli* (calculated in each 50 kb sliding window).

Our study focuses on the genomes of two seahorse species, the big-belly seahorse, *Hippocampus abdominalis* and the lined seahorse, *Hippocampus erectus*, and two outgroup species within the Syngnathidae, the Gulf pipefish, *Syngnathus scovelli*, and common seadragon, *Phyllopteryx taeniolatus* (fig. 1*A*). We recently generated a high-quality chromosome-level genome assembly of a male big-belly seahorse (He, et al. 2021). Using this as a reference, we newly produced and analyzed genomic and transcriptomic data from both sexes of this species, and those from its related species, the lined seahorse. Here, we now 1) identify the SDR of the two seahorse species, and show that at least one of them specifically evolved within the seahorse lineage, 2) describe evidence that both seahorse species have large regions showing Y-linkage, and investigate how these regions’ low recombination rates evolved, and 3) test whether these Y-linked regions have undergone genetic degeneration and/or adaptive changes, including whether SA selection is suggested. This test is based on the prediction that seahorse Y-linked regions should become enriched with male-specific pouch–expressed genes. Acquisition of such genes may involve movements from other chromosomes, probably requiring a long evolutionary time. If, however, a Y-linked region carrying many genes evolved recently, as in the case of the sex chromosome of the big-belly seahorse shown here, we might expect to detect an earlier stage of adaptation, in which pouch-expressed genes that are located in the region start evolving higher expression.

## Results

### Chromosome Evolution of Seahorse and Gulf Pipefish Species

We first describe our new results establishing that both seahorse species studied have extensive sex-linked regions that include many genes. We also evaluate the extent of sex-linkage and the evidence that it evolved recently in at least one of the seahorse lineages represented by our study species. Little was previously known about seahorse sex chromosomes, though a cytogenetic study suggested that the lined seahorse (*H. erectus*) might have a ZW sex chromosome pair (Liu, et al. 2020), which our results below do not support.

Cytogenetic studies reported diploid chromosome numbers ranging from 2*n* = 44 (in *H. erectus* and the Gulf pipefish, *S. scovelli*) to 2*n* = 58 among syngnathids, with most seahorse chromosomes being acrocentric, while those of Gulf pipefish are (sub)metacentric (Vitturi, et al. 1998; Liu, et al. 2020). The teleost ancestor was inferred to have 24 pairs of acrocentric chromosomes (Ohno 1974; Vitturi, et al. 1995; Mank and Avise 2006; Yoshida and Kitano 2021) suggesting two chromosome fusion or translocation events in the Syngnathidae lineage, as already suggested for the Gulf pipefish (Small, et al. 2016). In *H. abdominalis*, over 99% of the haploid genome is assembled into 21 chromosomes (He, et al. 2021), indicating one additional fusion or translocation event specific to *H. abdominalis* (supplementary fig. S1, Supplementary Material online). Most *H. abdominalis* chromosomes each show one-to-one sequence homology with a single *S. scovelli* or Nile tilapia chromosome, but distinct parts of Chr11 are homologous to separate *S. scovelli* chromosomes, LG15 and LG17 (fig. 1*B*) and Nile tilapia (fig. 1*C*). This suggests a chromosome fusion after the split from *H. erectus*, which is estimated to have occurred 11.4 Ma (He, et al. 2021).

Sequences of the *H. abdominalis* Chr6 (which, as shown below, is this species’ sex chromosome pair) can be aligned to two chromosomes of the distantly related non-teleost fish, *Lepisosteus oculatus* (the spotted gar, more details below in fig. 3*D*). This rearrangement is additional to the two chromosome fusion or translocation events within the Syngnathidae lineage (see above). It probably occurred before the teleost whole genome duplication and species radiation (Bian, et al. 2016), since homologs of these two gar chromosomes are also fused in many other teleosts, including zebrafish (Howe, et al. 2013), stickleback (Braasch, et al. 2016), arowana (Bian, et al. 2016), medaka, and the guppy, in which the fused chromosome is the one named LG16 (supplementary fig. S2, Supplementary Material online).

Compared with *S. scovelli*, the *H. abdominalis* genome is 44.8% larger, and all three seahorse species analyzed have higher repeat contents (1.38-fold for *H. erectus,* 2.07-fold for *H. abdominalis*, and 1.40-fold for *Hippocampus comes*, the tiger-tail seahorse, see supplementary fig. S3, Supplementary Material online). This indicates *Hippocampus*-specific expansions of several transposable element (TE) families (supplementary Table S2, Supplementary Material online), especially the DNA transposon TcMar-Tigger, Long Terminal Repeat (LTR) Ngaro subfamilies (fig. 1*A*), as well as two subfamilies of short interspersed nuclear element (SINE) tRNA-Core-L2 and DNA transposon Zator, which are both concentrated at the chromosome fusion junction in the middle of Chr11 of *H. abdominalis* (fig. 1*D*). About 55.2% and 15.9% of the entire genome's tRNA-Core-L2 and Zator sequences, respectively, are within this junction region (supplementary Table S3, Supplementary Material online). These two repeats are more abundant in the *H. abdominals* genome than in *H. erectus* (1.48 and 2.12 times higher, respectively; supplementary Table S4, Supplementary Material online), suggesting that they have recently expanded in *H. abdominals*. As we did not detect any telomeric repeats at the junction site, the tRNA-Core-L2 and Zator repeats may mark *H. abdominalis* centromeric regions.

The same two transposon sequences were indeed enriched at one end of 18 of the other 20 *H. abdominalis* chromosomes (supplementary Table S5, Supplementary Material online). Centromeric repeats commonly differ between species (Melters, et al. 2013), and the sequences identified here did not show homology with centromeric repeats reported in other teleosts. By examining regions homologous to the fusion junction site of *H. abdominalis* Chr11, we also identified putative centromeric repeats in *H. erectus* and *S. scovelli*. They are also enriched at the ends of the two chromosomes corresponding to linkage groups LG15 and LG17 of *S. scovelli* (supplementary Table S6, Supplementary Material online, fig. 1*D*), supporting the centric fusion hypothesis outlined above for *H. abdominals* Chr11.

### Chromosome-wide Recombination Patterns in Seahorses

To initially assess recombination landscapes before searching for sex-linked regions in our seahorse genomes, we describe features of the chromosome sequences of *H. abdominalis*, *H. erectus* (Lin, et al. 2017), and *S. scovelli* (Small, et al. 2016) that relate to recombination rates. First, the chromosomes exhibit spikes of repeat content at the inferred centromeric ends in all three species (Vitturi, et al. 1998; Liu, et al. 2020). The chromosomes also display pronounced patterns in GC content: based on the centromeric positions inferred above, the opposite ends of the chromosomes (which we term “tip regions”) show spikes of GC content in both seahorse genome assemblies (fig. 1*E*, *P* < 0.001, by Mann–Whitney *U* tests), resembling findings in many other teleosts (Costantini, et al. 2007; Matoulek, et al. 2020; Cheng, et al. 2021). The tip regions with GC spikes identified by change-point analyses of GC content within each chromosome (see Materials and Methods and supplementary fig. S4, Supplementary Material online) occupy 4.93% of the entire genome. This probably reflects GC-biased gene conversion (gGBC) associated with highly localized crossovers (Arbeithuber, et al. 2015) at one chromosome end in at least one sex, as suggested for the guppy, in which crossover rates in males correlate with intronic GC values (Charlesworth, et al. 2020). Correspondingly, GC contents of pericentromeric regions of each seahorse chromosome (defined as 3 Mb regions flanking the putative centromeric marks in fig. 1*D*) are significantly lower than in the rest of the respective chromosomes (*P* < 2.2e-16, Wilcoxon test, supplementary fig. S5, Supplementary Material online). These regions account for at least 18% of the *H. abdominalis* genome. Chromosome-wide GC patterns are much less clear in *S. scovelli*. Based on the centromeric positions inferred above in *S. scovelli*, (supplementary fig. S6, Supplementary Material online, supplementary Table S5, Supplementary Material online) 8 of its 22 chromosomes are inferred to be metacentric, submetacentric or subtelocentric, and intrachromosomal rearrangements may have obscured the overall GC patterns.

No dense genetic maps are yet available for seahorses. However, sex-averaged recombination rates (ρ values), estimated by analyzing linkage disequilibrium across *H. abdominalis* chromosomes (using 30 males and 29 females from a cultured population, see Materials and Methods), show the expected tendency to be highest in regions with high GC content (Pearson correlation *r* = 0.35, *P* < 2.2e-16; supplementary fig. S7, Supplementary Material online), consistent with high meiotic crossover rates in those regions, and low rates in other regions in one or both sexes.

Although direct evidence from sex-specific genetic mapping data is not yet available in the seahorse species studied here, independent evidence supports the conclusion that strong crossover localization to small chromosome end regions occurs specifically in males. Genetic mapping of microsatellite markers estimated a 10-fold higher recombination rate in females than in males in the Western Australian seahorse, *Hippocampus angustus* (Jones, et al. 1998). This suggests that, in males, recombining regions are physically small, so that most markers will be in large regions that rarely cross over.

### Sex-linked Regions of Three Syngnathidae Species

To identify the regions that include the sex-determining genes, we sequenced the genomes of 30 and 10 individuals, respectively, of each sex of captive populations of *H. abdominalis* and *H. erectus* at around 15× coverage per individual. Since no chromosomal assembly of *H. erectus* was available, we assembled the *H. erectus* sequences using RaGOO (Alonge, et al. 2019) to order and orient scaffolds into chromosomes using the *H. abdominalis* genome assembly as a reference.

Given the known great diversity of teleost sex-linked regions (Kottler and Schartl 2018; El Taher, et al. 2021; Pan, Feron, et al. 2021a), and the absence of prior information from seahorses, other than that sex chromosome hetermorphism has not been detected (Liu, et al. 2020; Feron, et al. 2021), we combined multiple approaches to search for completely sex-linked regions. These include comparing males and females for genomic read coverage and sequence diversity (or SNP density) and estimating F_ST_ between the sexes. If a Y- or W-linked region has become highly degenerated, the genomic coverage of the X- or Z-linked region should be twice as high in the homogametic as the heterogametic sex, and Y- or W-specific sequences should have coverage close to zero in the homogametic sex. SNP density and F_ST_ between the sexes can potentially detect less degenerated fully sex-linked regions, which may nevertheless include many sex-specific variants, especially if the regions are extensive, while genome-wide association study (GWAS) treating sex as a binary trait can detect the sex-linked regions whose sequences are even less differentiated, or are physically small, given a sufficiently high density of sequence variants (Palmer, et al. 2019; Vicoso 2019).

Neither of the two seahorse species nor the Gulf pipefish, exhibits a pronounced sex difference in coverage or SNP density in any chromosomal region, except for the highly repetitive fusion site in Chr11 in *H. abdominalis* (supplementary fig. S8–14, Supplementary Material online). Their sex-linked regions may have originated too recently for genetic degeneration to have led to major gene loss, or for extensive sequence divergence to have evolved. Although accurate read mapping can be hindered by polymorphic repetitive elements within Y-linked regions, this should lead to low coverage in males, and is unlikely to lead to falsely suggest a recent origin. Also, neither seahorse species has any genomic region whose read coverage suggests a sex-specific duplication. The *AMHR2* gene has evolved by such duplication to be a male-determiner (termed *AMHR2Y*) in several other teleosts (Herpin and Schartl 2015; Pan, et al. 2016), and a duplicate putatively sex-determining *AMHR2Y* gene is found in two other syngnathids (Qu, et al. 2021). In both seahorse species, however, this gene has equal coverage in both sexes (supplementary fig. S15, Supplementary Material online), consistent with the previous conclusion that it is absent from the seahorse lineage (fig. 1*A*) (Qu, et al. 2021). A previous GWAS based on RAD-seq data also failed to identify any sex-linked region in *H. abdominalis* (Feron, et al. 2021).

**Fig. 2.**
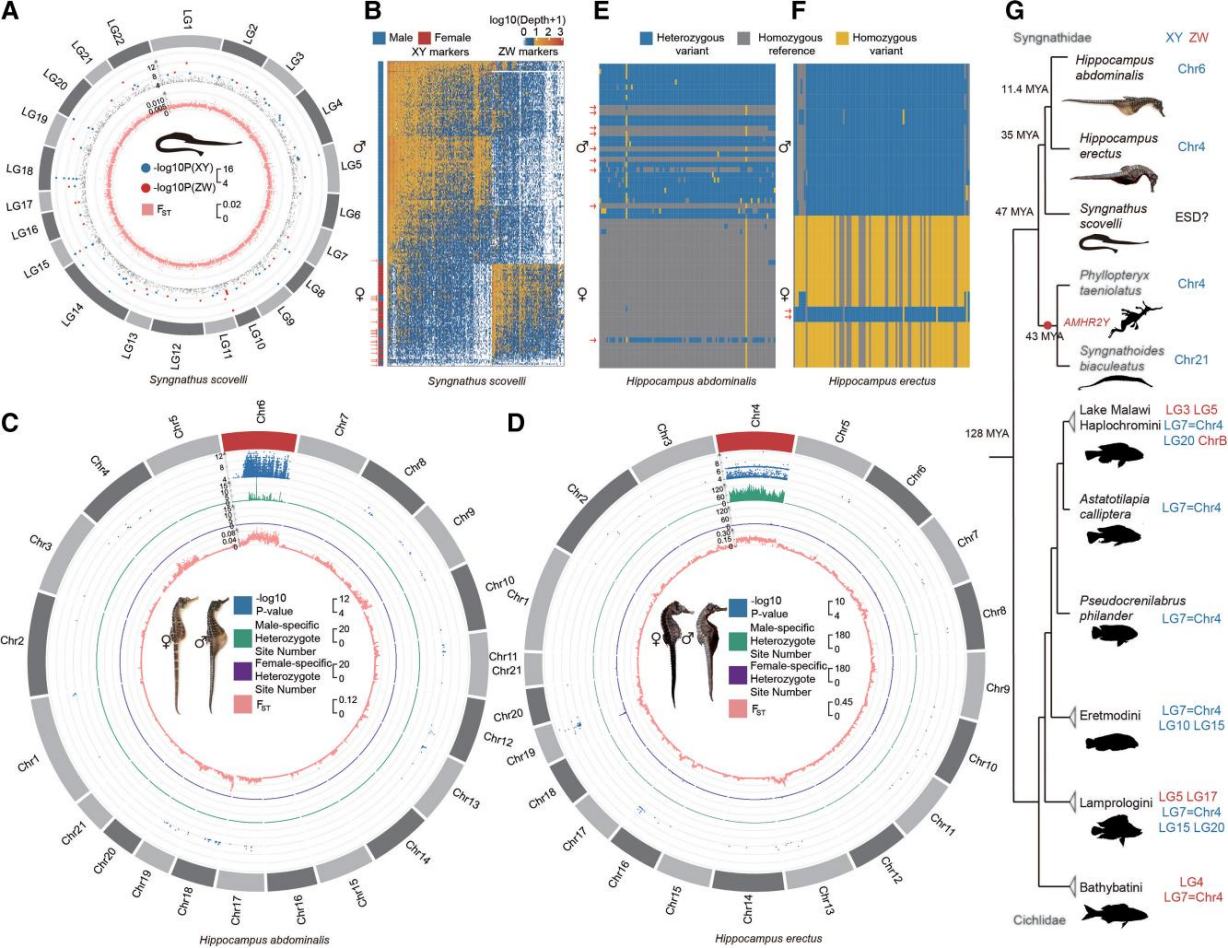
Independent evolution of sex-linked regions of Syngnathid species. (*A*) Sex-associated RAD markers in *Syngnathus scovelli*. Markers significantly associated with sex are labeled based on male or female heterogamety in the outer track of the circos plot. F_ST_ values between males and females are shown in the inner track. Neither suggests a sex chromosome. (*B*) Heatmap showing individual sequencing depth for all markers present in at least five *S. scovelli* individuals. Each column corresponds to a marker, and each row corresponds to one male or female individual. Males and females cluster in separate groups, except for 1 female and 19 male outliers marked by arrows. (*C* and *D*) Sex-linked regions of *Hippocampus abdominalis* and *Hippocampus erectus.* The figure shows various metrics calculated in 50 kb sliding windows across each chromosome, with the first tracks for the –log10 *P*-values of GWAS tests shown (for sex-associated SNPs with *P* < 10^−4^ in *H. abdominalis* and *H. erectus*), and the second tracks for the numbers of sites that are heterozygous in males and homozygous in females after excluding the possible sex-reversed individuals, while the third tracks show the sites that are heterozygous in females and homozygous in males, and the innermost tracks show the mean F_ST_ value between the sexes. Both the GWAS results and increased F_ST_ values suggest Y-linked regions occupying large parts of the *H. abdominalis* chromosome 6 and the *H. erectus* chromosome 4. (*E* and *F*) Genotypes of the 100 most strongly associated SNPs with sex in the GWAS analyses of *H. abdominalis* and *H. erectus* (ranked by their *P*-values). Each row corresponds to one male or female individual, and each column corresponds to a SNP within the sex-linked region. In both species, males were mostly heterozygous and females were mostly homozygous at these sites, indicating male heterogamety. Individuals with the inferred genotype of the opposite sex at all these sites are indicated by arrows. (*G*) Phylogenetic tree and sex chromosome of Syngnathidae and the more distant outgroup Cichlidae species. Sex chromosomes of Syngnathidae and Cichlidae species are colored to indicate XY (blue) and ZW (red) sex-determining systems.

In contrast to the approaches just outlined, GWAS results using whole genome resequencing data of individuals from captive populations (see Materials and Methods) did detect sex-linked regions in both seahorse species studied here, *H. abdominalis* and *H. erectus*. Indeed, significantly sex-associated SNPs are found across about two-thirds of the *H. abdominalis* Chr6, based on 30 individuals of each sex from the same family (fig. 2*C*, supplementary fig. S16, Supplementary Material online), and almost the entire *H. erectus* Chr4, based on 10 individuals of each sex (fig. 2*D*). We denote these chromosomes by HaChr6 and HeChr4, respectively. In the regions just mentioned, 82% of *H. abdominalis* sex-associated SNPs are heterozygous in most, though not all, males examined (see below), and homozygous in most females (fig. 2*E*–*F*). In *H. erectus*, 89.3% of the sex-associated SNPs are heterozygous in most males and homozygous in most females. None of the sex-associated SNPs in *H. abdominalis*, and only 0.4% in *H. erectus* shows higher frequencies of heterozygotes in females. The concentration within large contiguous chromosome regions of sites that are heterozygous much more often in males than females indicates that these extensive regions are either fully or partially, but closely, linked to the sex-determining loci in these seahorses.

In the same captive samples of both species, the candidate sex-linked regions exhibit higher differentiation between the sexes (measured by F_ST_) than the rest of the chromosome, or other chromosomes (*P* < 0.001, by Mann–Whitney *U* tests) (fig. 2*C*), and, importantly, heterozygote frequencies are higher in males than females, as detected by negative F_IS_ values across the sex-differentiated region (*P* < 0.001 by Mann–Whitney *U* tests, see supplementary figs. S17–18, Supplementary Material online). These results all support male heterogamety with extensive Y-linked regions in both species. Although some other genome regions also show high F_ST_ values between the sexes (including part of *H. erectus* Chr13, supplementary fig. S19, Supplementary Material online), only the candidate sex-linked regions on HeChr4 and HaChr6 are supported by GWAS analyses. The elevated F_ST_ between the sexes, and strongly negative F_IS_ specific to the differentiated region, are especially clear in HeChr4. We conclude that the previously suggested ZW system in *H. erectus* (Liu, et al. 2020) is not correct, at least for our material (it is possible that heterogamety might vary, as in the platyfish or *Gambusia* species (Black and Howell 1979; Volff and Schartl 2001). Overall, these results, together with those in the previous section, suggest that both seahorse species studied have highly similar, homomorphic sex chromosomes, or physically small completely sex-linked regions. Therefore, either complete sex-linkage evolved recently or these regions recombine often enough to prevent Y chromosome divergence (see below).

We also analyzed the published restriction site-associated DNA sequencing (RAD-Seq) data with on average 9.5× sequencing coverage per individual from 167 male and 57 female *S. scovelli* individuals from a wild population (Flanagan and Jones 2017). Neither GWAS nor F_ST_ analyses, nor analysis of coverage, identified any contiguous sex-linked region (fig. 2*A*, supplementary fig. S11, Supplementary Material online), and GWAS analysis of the RAD-seq data did not identify the heterogametic sex (fig. 2*A*, supplementary fig. S20, Supplementary Material online): among the 419 sites that were significantly associated with sex in the natural population studied, with *P* < 0.05 by χ^2^ tests, 248 sites were heterozygous only in males, suggesting male heterogamety, but 171 suggested female heterogamety (fig. 2*B*). These variants are distributed across many different chromosomes. RAD-seq variants are probably too sparse for inferring the actual sex-determining locus. These data, together with the previously reported female-biased sex-ratios (Bolland and Boettcher 2005) in a wild *S. scovelli* population suggest that *S. scovelli* might have environmental sex determination (ESD), though this remains uncertain.

### Sex Chromosome Turnover(s) in Seahorses

An autosome must have evolved into a sex chromosome in one or both of the two seahorse species studied. Two outgroup species, *P. taeniolatus* and *Syngnathus biaculeatus*, also have XY systems (Qu, et al. 2021). The Syngnathidae ancestor could therefore either have had an XY system or ESD. The homolog of Chr4, with the inferred Y-linked region in *H. erectus*, is also a sex chromosome in several cichlid species (El Taher, et al. 2021), and in *P. taeniolatus*. However, it is unlikely that *H. erectus* inherited it as a sex chromosome from a syngnathid ancestor, because as mentioned above, both Syngnathidae species so far studied carry the *AMHR2Y* duplication, but the HeChr4 chromosome does not, and must carry a different male-determining gene. A turnover in *P. taeniolatus* and *S. biaculeatus* lineage that includes the two species with the *AMHR2Y* duplication cannot currently be excluded. Among the cichlid species that were shown to have the homologous sex chromosome (numbered LG07 in cichlids, fig. 1*C*), some species have ZW sex systems, and Chr4 is likely to have become a sex chromosomes convergently in different tribes (El Taher, et al. 2021). Taken together, the data show that the Y-linked region on chr6 of *H. abdominalis*, and probably also that on chr4 of *H. erectus* must have evolved recently, after their divergence from the common ancestor of seahorses.

### Defining the Boundaries Between Sex-linked Regions and the Adjacent Pseudoautosomal Regions (PARs)

**Fig. 3.**
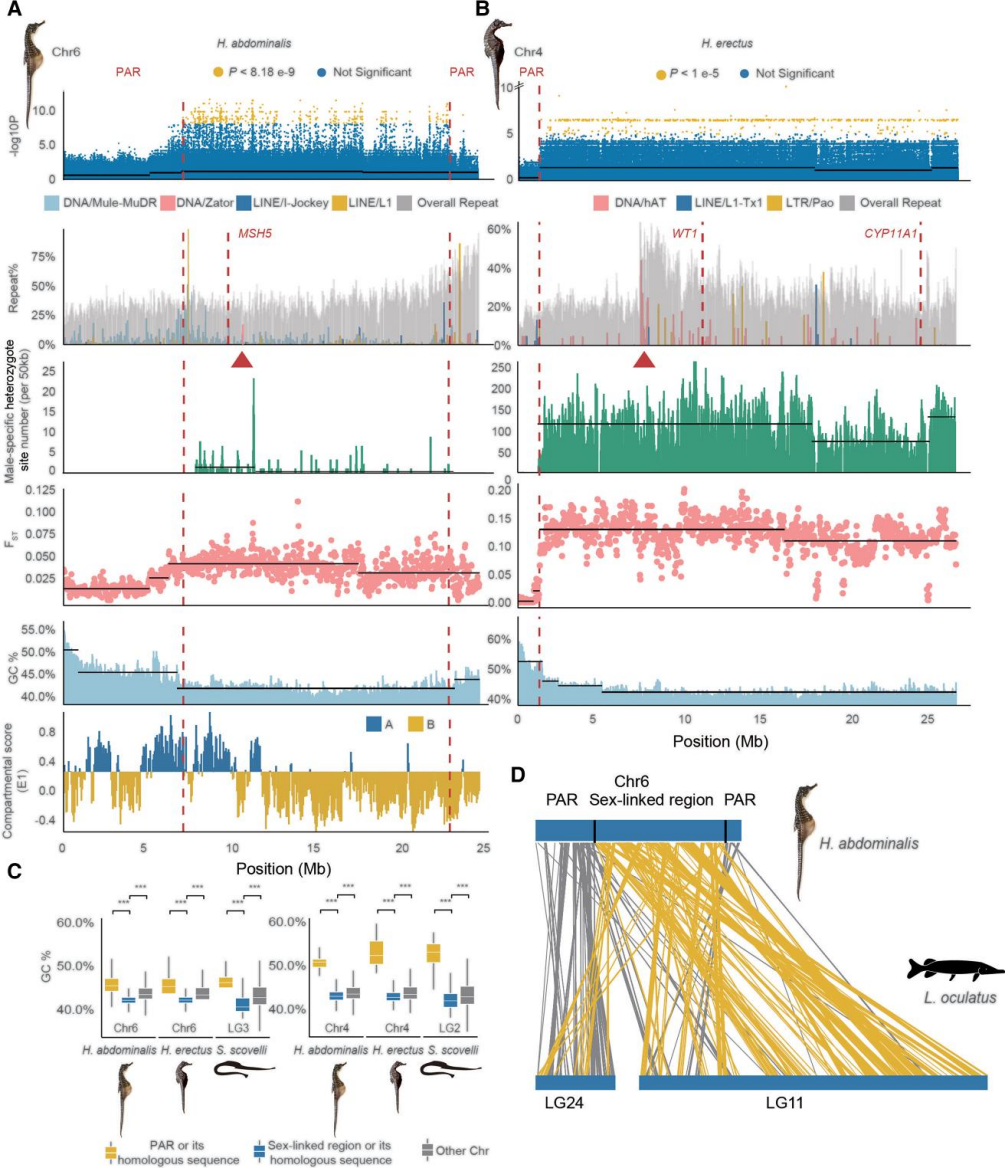
Repeat and putative recombination landscapes at the seahorse sex-linked regions. (*A*) Part of the sex-linked region inferred on the *Hippocampus abdominalis* Chr6 (7.03–22.68 Mb). The first track shows the sex-associated SNPs identified by our GWAS; the second track shows the overall repeat content, with the inferred centromeric region (based on enrichment of the DNA element Zator, see fig. 1) labeled by a triangle, and the candidate sex–determining gene *MSH5* indicated with a dashed line; the third track shows the numbers of sites per 50 kb that are heterozygous in males but homozygous in females, after excluding the possibly sexually reversed individuals; the fourth track shows the mean F_ST_ values between males and females in 50 kb windows, and the fifth track shows the GC content values; the last track shows the A/B or active/repressive compartment distribution inferred from Hi-C data. (*B*) Part of the sex-linked region of *Hippocampus erectus* Chr4 inferred based on a high density of sex-associated SNPs in our GWAS (1.28–26.1 Mb). The centromeric region was inferred from enrichment of the DNA element hAT (red triangle), and the candidate sex–determining genes *WT1* and *CYP11A1* are indicated by dashed lines. Dashed vertical lines also label the boundary between the putatively sex-linked regions and the PAR. Horizontal black lines indicate the different mean values of each quantity inferred by change-point analysis. (*C*) GC content of the HeChr4 and HaChr6 sex-linked regions. (*D*) Syntenic regions in HaChr6 of *H. abdominalis* and LG24 and Chr11 of *Lepisosteus oculatus*, the spotted gar. Homologous sequences located within the sex-linked region of *H. abdominalis* are shown with yellow lines, while gray lines show those in the PAR.

We next describe further genomic evidence in support of the idea above that the seahorse sex chromosomes have large regions that recombine rarely, and small pseudoautosomal regions. Based on the locations enriched with the centromeric repeat marks noted above (the DNA transposons Zator in *H. abdominalis*, and hAT and LTR Pao in *H. erectus*, fig. 1*D*, supplementary fig. S21, Supplementary Material online), we inferred that the HaChr6 and HeChr4 sex chromosomes are metacentric or submetacentric, like their autosomal homologs in the other species studied (fig. 1*D*, supplementary fig. S21, Supplementary Material online). Within HaChr6 and HeChr4, change-point analyses using 50 kb sliding windows identified significant changes between adjacent regions in the between-sex F_ST_ values, or using the *P*-values of our GWAS analyses, or the densities of heterozygous sites in males (fig. 3 and supplementary fig. S16, Supplementary Material online). These significant changepoints allow us to define the boundaries between regions with different strengths of sex-linkage. In *H. erectus,* a single clearly defined PAR was inferred, occupying only 1.28 Mb (4.9% of the HeChr4 length); a small and very fragmented region at the other chromosome end is homologous to the high GC region of the homologous *H. abdominalis* chromosome, and could be a second PAR (supplementary fig. S22, Supplementary Material online). As HaChr6 is derived via an ancient centric fusion between two chromosomes, two PARs might be expected, because the evolution of sex-linkage on one arm might not be expected to lead to suppressed recombination on the other arm. We indeed found a PAR at each end of HaChr6 (fig. 3*A*–*B*). GWAS analysis suggests two possible PARs (7.04 and 1.79 Mb long, respectively) together occupying 8.83 Mb in total (36.1% of HaChr6), but the transitions in F_ST_ and density of male heterozygotes defining them (and especially the right-hand one, on the arm derived from LG11 of *L. oculatus*, see fig. 3*D*), are not as sharp as the one defining the *H. erectus* PAR boundary.

The regions inferred as “non-PAR” probably still recombine, at least occasionally, as associations between genotypes of the sex-linked variable sites and phenotypic sex (based on brood pouch development in mature fish) were incomplete in both species (some apparently male homozygotes or female heterozygotes were found within the putatively sex-linked regions; fig. 2*E*–*F*). This might reflect sex-reversals, as reported in European tree frogs (Stöck, et al. 2011), and in the wild in a seahorse species *Hippocampus reidi* (Freret-Meurer, et al. 2021). We genotyped 10 individuals of each phenotypic sex for 10 randomly picked *H. abdominalis* sex-associated SNP sites scattered within the candidate completely sex-linked region detected by our initial GWAS analyses. Seven tested sites were heterozygous more often in males than in females (supplementary fig. S23, Supplementary Material online, supplementary Table S7–8, Supplementary Material online), but three were homozygous in all 20 *H. abdominalis* individuals tested, of both sexes. Among 59 *H. abdominalis* individuals and 20 *H. erectus* individuals from captive populations whose whole genomes were sequenced in this study (see Materials and Methods), 15% and 10% of the individuals, respectively, had genotypes expected for the sex other than their phenotypic sex at almost all polymorphic sites ascertained as showing sex-associations (fig. 2*E*–*F*). Sex-linkage is therefore not complete in either sex.

Despite the physically large sizes of the apparently sex-linked regions of HeChr4 and especially HaChr6, their sex-determining systems (especially in *H. erectus*) could have evolved recently. This can be further tested in the future by studying other more closely related seahorse species, such as the sister species of *H. erectus*, *H. hippocampus* (Li, et al. 2021). The sex-linked regions of either or both species studied here could either have evolved as a direct consequence of ancestral crossover localization (as proposed in the guppy (Bergero, et al. 2019)), or it could have evolved subsequently. In either case, given enough time, this would allow evolution of sequence variants associated with the male-determining allele (linkage disequilibrium, or LD). If recombination was not completely suppressed, Y–X divergence would remain low, and genetic degeneration would not occur, but F_ST_ between the sexes could be higher than elsewhere in the genomes, as it reflects LD (Charlesworth, et al. 1997).

### Ancestral Low Recombination Rates and Seahorse Sex Chromosome Evolution

In both *H. abdominalis* HaChr6 and *H. erectus* HeChr4, the centromere positions inferred above are consistent with (peri)centromeric regions being within the sex-linked regions (fig. 3*A*–*B*). This is also supported by the fact that, in chromatin interaction data from *H. abdominalis* (He, et al. 2021), 76.9% of the HaCh6 sex-linked region is classified as repressive chromatin domains (the B compartment in fig. 3*A*); as expected, A compartment genes have significantly higher expression (e.g. in brain and testis) than B compartment ones (*P* < 2.2e-16, Mann–Whitney *U* tests). The sex-determining genes of both seahorse species may therefore have evolved within pericentromeric regions with ancestrally low recombination rates. The autosomal regions homologous to each of these regions in the other seahorse species and the Gulf pipefish also have significantly lower GC content than other genome regions, consistent with repressive chromatin states being established before these regions became sex-linked (*P* < 0.001 by Mann–Whitney *U* tests, fig. 3*C*).

We next asked whether the PAR of either species was formerly larger, and has become smaller by chromosome rearrangements, enlarging the ancestrally sex-linked regions. We searched the PAR boundaries and their flanking regions for evidence of inversions that might have suppressed recombination, by examining the paired-end relationships of Illumina reads and male PacBio reads of both species spanning the regions but found no evidence for such rearrangements (see Materials and Methods, supplementary fig. S24–S25, Supplementary Material online). Alignment between HaCh6 and the homologous LG3 of Gulf pipefish also did not reveal inversions at the PAR boundary (supplementary fig. S26, Supplementary Material online). Within these species’ sex-linked regions, we also did not detect any signatures of new evolutionary strata; there are no consistent regional differences in male–female F_ST_ values, nor in the numbers of sites that are predominantly heterozygous in males but homozygous in females (fig. 3*A*–*B*, supplementary fig. S16, Supplementary Material online). Based on sex-linked genes in which all variable sites are heterozygous in all *H. abdominalis* males but homozygous in females (1.12% of the sex-linked genes), and after excluding individuals that likely have undergone sex-reversals (fig. 2*E*–*F*), we estimated the average synonymous site divergence between the X and Y (K_s_) to be 0.002; in *H. erectus* the corresponding K_s_ estimate was 0.006 (in which 423, or 41.6%, of the sex-linked genes satisfied this criterion). As this must over-estimate the divergence, the very low values indicate that both species’ sex-linked regions either originated very recently or, more likely, based on the evidence above, still recombine, albeit at a very low rate. Even in HaChr6, in which F_ST_ between the sexes is quite low (fig. 3*A*), significant changes in F_ST_ and GC content coincide, supporting the conclusion that their recombination rates have been different over considerable evolutionary times, and thus our classification into PAR versus MSY regions.

One of the *H. abdominalis* PAR boundaries identified above coincides with an ancient chromosome fusion site (fig. 3*D*), suggesting that the sex-linked region evolved near an ancestral centromere created in a centric fusion event. The HaChr6 sex-linked region represents the larger of two ancient fish microchromosomes (the homolog of the spotted gar LG11), and the PAR represents the smaller one (homologous with the spotted gar LG24) involved in the fusion, with the centromeric region separating them (fig. 3*D*). The chromosome arm that evolved a male-determining factor might already have been largely non-recombining in males, or may have stopped recombining subsequently, but the other arm could have remained unaffected and would be unlikely to stop recombining by a pericentric inversion, as these are rare events (although, as mentioned in the Introduction, recombination suppression can evolve without an inversion). Selection may not have favored suppressed recombination on this arm, perhaps because no SA polymorphisms became established in this small chromosome arm in the short evolutionary time since HaChr6 became a sex chromosome.

Different TE subfamilies are specifically enriched at the PAR boundaries of both seahorse species studied, compared with their genome averages. In *H. abdominalis*, long interspersed nuclear element (LINE) L1 elements showed a 6.82-fold enrichment, and the DNA transposon Mule-MuDRs showed a 6.03-fold enrichment. In *H. erectus*, LINE/L1-Tx1 sequences were 6.67-fold enriched at the PAR boundary (fig. 3*A*–*B*). However, analyses of Illumina reads spanning the TE insertions yielded no evidence that they are male-specific. In the other seahorse and Gulf pipefish species, neither LINE/L1 nor LINE/L1-Tx1 elements were enriched in the homologous regions (supplementary fig. S27, Supplementary Material online), suggesting that these are recent species-specific insertions of these TEs.

### Candidate Sex-Differentiation Genes in the two Seahorse Species

The different locations of the sex-linked regions in the two seahorse species (fig. 2*D*) implies independent acquisition of a male-determining gene in at least one species, and parts of HeChr4 and HaChr6 must have been evolving under at least partial sex-linkage for different evolutionary times. These regions may have become enriched for genes participating in gonadogenesis and gonad differentiation, and, as mentioned in the introduction, changes in expression of genes in the region with sex-related functions, including in the male pouch, might also evolve after their male-determining factors evolved (Ellegren and Parsch 2007). To identify genes of these kinds, we searched gonad transcripts from *H. abdominalis* and *H. erectus* (supplementary fig. S28, Supplementary Material online; see Materials and Methods) for genes transcribed at different levels in gonads of either sex compared with other tissues, or whose transcription patterns have changed compared with their autosomal orthologs in the other seahorse or Gulf pipefish species. As it is unclear exactly when in development sex determination is initiated in seahorse species, we sampled tissues spanning different developmental stages, from the time when gonads first become morphologically visible until they become distinctly different in the two sexes. The genes described below are therefore more likely to function in sex-differentiation processes than in sex determination, especially if they are only transiently expressed in one sex. Nevertheless, sex differences in expression of the sex-determining genes sometimes extend to late developmental stages. For example, in amphibians *AMH* is predominantly expressed in differentiating testes, after the sex-determining stage (Pan, Kay, et al. 2021b).

**Fig. 4.**
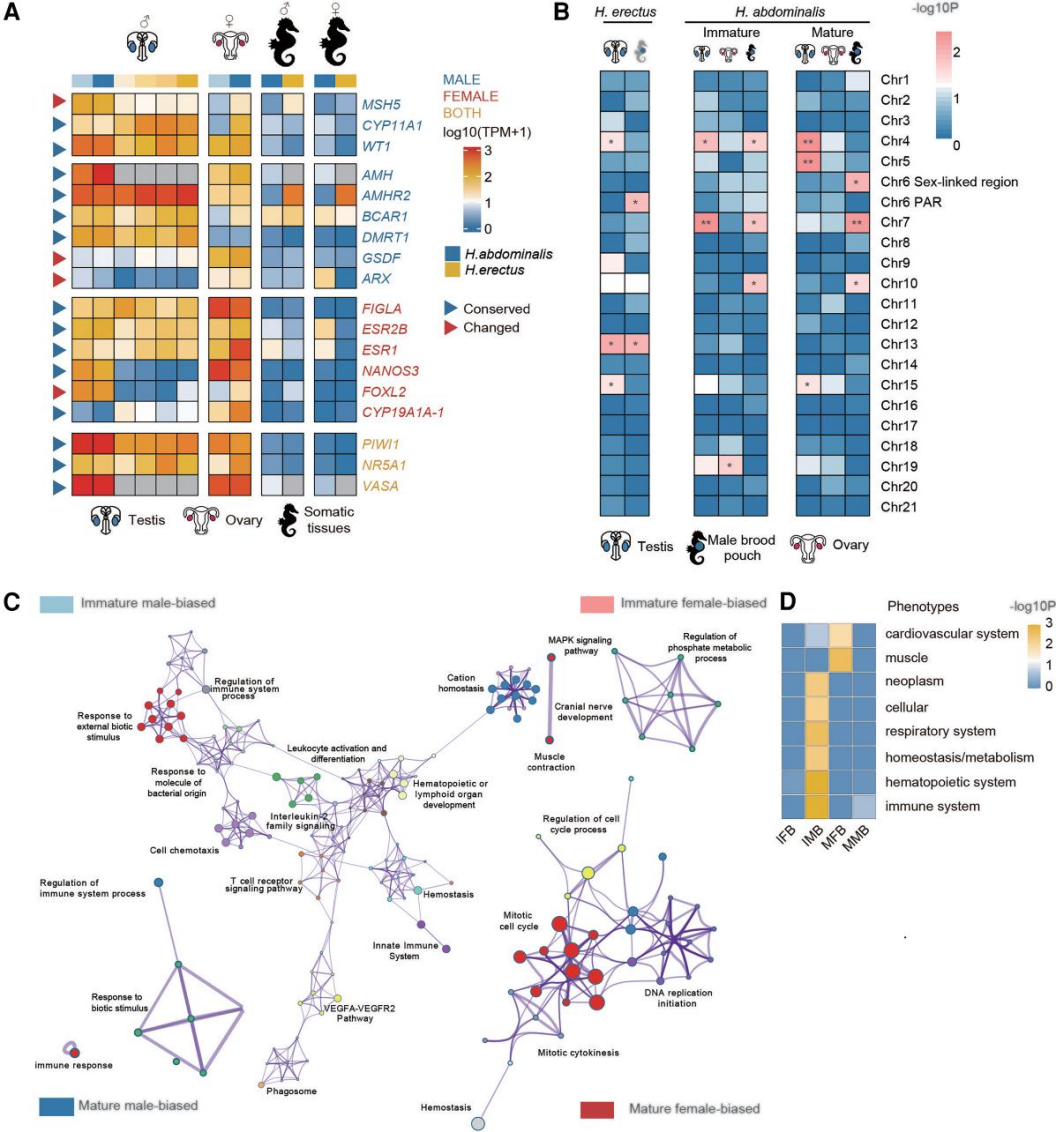
Evolution of sex-related genes associated with the independent sex chromosome evolution. (*A*) Heatmap showing the transcription patterns of putative sex-determining genes and gonad development genes in different tissues of *Hippocampus abdominalis* (blue) and *Hippocampus erectus* (yellow). Darker colors in the first row represent later developmental stages. Different intensities of blue indicate the number of days after fertilization (DAF), from 90 (light) to 150 (deep), while yellow intensities indicate later stages, 150 DAF (lightest), 210 DAF, 240 DAF (pregnant) and 240 DAF (post-pregnant, deepest). Gray boxes represent missing orthologs in *H. erectus*. Genes are labeled with different colors: blue for male-determining or testis development genes, red for female-determining or ovary development genes and yellow for genes with functions in both sexes. Blue triangles represent genes whose gonad transcription patterns are similar to those of their orthologs in the other seahorse or a teleost species, and red triangles represent genes whose gonad transcription patterns differ between the species. The suffix “−1” added to the gene name indicates that it has multiple copies in the genome. (*B*) Enrichment on different chromosomes of tissue-biased genes (relative to male muscle, brain, and kidney tissues) and male pouch-biased genes (relative to female abdominal skin). Heatmap showing the scaled log10 *P*-values of Fisher's tests for chromosomal enrichment. Chromosomes significantly enriched for genes with higher levels in pouch relative to other male tissues are labeled with asterisks (Fisher's Exact test, *P < 0.05, **P < 0.01). (*C*) GO enrichment of different categories of genes. Immature male-based: genes that have higher transcription levels in the immature (3 months old) than the mature (5 months old) pouch, and also than in the female abdominal skin at the same stage. Immature female-biased: genes that have a higher transcription level in the immature (3 months old) than the mature pouch (5 months old), but a higher transcription level in the female abdominal skin than the pouch etc. Each node represents one enriched GO term. Nodes of related GO functional terms are shown in similar colors, and the widths of the lines connecting the nodes indicate the similarity between the terms, while the node sizes represent the percentage of input genes belonging to each term. (*D*) Enrichment analysis of homologous mouse genes’ mutant phenotypes for different types of *H. abdominalis* genes, with a heatmap indicating the *P*-values of Bonferroni corrected Fisher's Exact tests. Immune system–related phenotypes were enriched in both immature and mature male-biased genes. IFB, immature female-biased; MFB, mature female-biased; IMB, immature male-biased; MMB, mature male-biased.

The HeChr4 sex-linked region includes 1,018 identified genes, one of which, *CYP11A1*, may act during male gonad differentiation. In both seahorse species, *CYP11A1* showed testis-biased transcription (relative to other male somatic tissues, including muscle, brain, kidney, and brood pouch). Its transcript levels increased during testis development and male gestation in *H. erectus*, consistent with an important male function having evolved in this species (supplementary fig. S29, Supplementary Material online). In *H. abdominalis*, this gene is not sex-linked, and the average expression level across different testis development stages was 5.38 times lower than in *H. erectus*, and transcript levels decreased during testis development (fig. 4*A*, supplementary fig. S29, Supplementary Material online). Its mammalian ortholog encodes a key steroidogenic enzyme (Morohashi, et al. 1993), and it was reported to have sex-biased transcription which changes during gonad development in other teleosts, including Nile tilapia and olive flounder (Ijiri, et al. 2008; Liang, et al. 2018). Another candidate male-determining gene is *WT1*, which was reported to be essential for mammalian male determination (Hossain and Saunders 2001), and showed higher transcript levels in *H. erectus* testis than somatic tissues. Its transcript level also increased during testis development and male gestation (supplementary fig. S29, Supplementary Material online), but was 2.5 times lower on average than in *H. abdominalis*.

The HaCh6 sex-linked region includes 624 identified genes, none of which is orthologous to known vertebrate sex-determining or sex-differentiation genes. This species’ sex determination may have arisen via a turnover event, as outlined above, and may involve a gene with no previously known function in gonad development, as was found for the rainbow trout male-determining gene *sdY* (Bertho, et al. 2018). One interesting sex-differentiation candidate gene is present, however; this is *MSH5*, whose mouse ortholog is required for proper meiosis (de Vries, et al. 1999) (mouse mutants show reduced gonad size and infertility in both sexes (Edelmann, et al. 1999). This gene is transcribed at a 47-fold higher level in the immature *H. abdominalis* testis and 24-fold higher in the mature testis, relative to ovary or somatic tissues at the same stage; the *H. erectus* ortholog shows no such bias, and had an average transcript level in testis 6.51 times lower than in *H. abdominalis* (fig. 4*A*, supplementary fig. S29, Supplementary Material online). *MSH5* is located close to the putative HaCh6 centromere.

Sex chromosome turnovers may also be followed by changes in the transcription of genes involved in the sex-differentiation pathway located elsewhere in the genome. To test this, we compiled a list of 72 genes previously reported to be involved in gonad development, sex determination, or gonad differentiation of other teleost species (Chen, et al. 2018; Guiguen, et al. 2018; Shen and Wang 2018; Taboada, et al. 2018) (fig. 4*A*, supplementary fig. S30, Supplementary Material online). Most of these genes are autosomal in both seahorse species studied here, and most showed gonad transcription patterns similar to those of their orthologs in the other seahorse species or other teleosts. For example, orthologs of the generally male-determining genes *DMRT1* and *AMH* showed testis-specific transcription in both seahorse species. The ortholog of *CYP19A*, whose protein product converts androgens to estrogens and plays an important role in female-determination in many other teleost species (Shen and Wang 2014), is specifically transcribed in the ovary of *H. abdominalis*. The orthologs of two germline genes, *VASA* and *PIWI*, are transcribed specifically in the gonads of both sexes in both seahorse species. Finally, a few genes had the highest transcript levels in the gonads of the opposite sexes in different species (fig. 4*A*).

However, we detected intriguing changes in some sex-differentiation pathway genes in *H. abdominalis* (fig. 4*A*), as might be expected given that a turnover must have occurred in this species (although the event is recent enough that changes might not have occurred). None of these genes is in the newly evolved sex-linked region of HaChr6. One example is *FOXL2*, a highly conserved gene that is predominantly transcribed in ovary granulosa cells of both mammals and some other teleosts (Bertho, et al. 2016), but shows testis-specific transcription in *H. abdominalis* but not *H. erectus*. In contrast, the *GSDF* gene, which is the male-determining gene in several other teleosts (Pan, et al. 2016) has become specifically transcribed in the ovary of *H. abdominalis*.

### Evolution of Gene Transcription in the Newly Evolved Sex-linked Region of *H. abdominalis*

Finally, to investigate possible changes in gene expression after genome regions newly became sex linked, we compared transcription patterns, particularly in the sex-specific organs (gonads and male brood pouch), in the two seahorse species. During its development, the pouch undergoes drastic tissue remodeling (Whittington, et al. 2015), with 4,505 genes (19.7% of all *H. abdominalis* annotated genes) changing their transcription between immature and mature pouch stages. The set of genes that were differentially expressed between these pouch stages was also enriched in genes with expression differences between male pouch and female epidermal tissue (representing the likely ancestral transcription state) in reproductively mature animals, using a threshold of at least twice the mean TPM (Transcripts Per Kilobase per Million reads) of the female tissue from specimens (*P* < 2.2e-16 by Fisher's Exact test; supplementary fig. S31, Supplementary Material online). We also defined 17 genes as pouch-biased, because they had higher expression than in other male tissues at the same developmental stage (muscle, brain, kidney). Eight of these showed higher transcript levels in the male pouch than in female abdominal epidermal tissues of *H. abdominalis*. We compared the representation of such genes, and genes transcribed at higher levels in mature male brood pouch tissue, relative to other tissues at the same developmental stage (muscle, brain, kidney), in different *H. abdominalis* genome regions against the genome-wide background by Fisher's Exact tests. This analysis suggested over-representation (*P* < 0.05, Fisher's Exact test) of such genes in the region of HaCh6 that recently became sex-linked (which includes 17 such genes), but not among HaCh6 PAR genes (though chromosome 7 also showed an enrichment in such genes, see fig. 4*B* and supplementary fig. S32, Supplementary Material online). Of the 13 autosomal orthologs of the 17 pouch-biased genes in *H. erectus*, 2 are pouch-biased, versus 11 that are not, again consistent with changes having evolved in the *H. abdominalis* genome (no orthologs were found on the homologous *H. erectus* chromosome for 4 of these genes). Thus some HaChr6 genes probably evolved increased expression in the male brood pouch after this *H. abdominalis* chromosome arm acquired the present male-determining gene, as expected for male-beneficial mutations.

Supporting the hypothesis that their male-biased transcription relates to pouch development, the *H. abdominalis* genes with pouch-biased expression are enriched for gene ontology (GO) terms including immune response (e.g., “T cell receptor signaling pathway”, “innate immune response”, “cellular homeostasis’ etc.) and hemopoiesis (‘leukocyte activation and differentiation”, “hematopoietic organ development” etc.; supplementary Table S9, Supplementary Material online), consistent with previous results showing that evolution of male pregnancy in seahorses is associated with turnovers of immune-related genes (Roth, et al. 2020). Genes with higher transcription levels in female epidermal tissue than male pouch tissue showed no such enriched GO terms (fig. 4*C*). Mutants of the mouse orthologs of these seahorse genes are also enriched in phenotypic defects in immune or hematopoietic systems (*P* < 0.01, Fisher's Exact test), while the female epidermal tissue-biased genes are not (fig. 4*D*, supplementary Table S10, Supplementary Material online).

The *H. erectus* HeChr4 sex-linked region is enriched for testis-biased genes. However, the enrichment probably pre-dates the divergence of these two species, as its autosomal homolog in *H. abdominalis* shows the same enrichment (*P* < 0.01 for both, regardless of different definitions of tissue-biased genes (see Materials and Methods and supplementary fig. S33, Supplementary Material online).

## Discussion

### Sex Chromosome Turnovers in Seahorses and the Origins of Their Extensive non-recombining Regions

Sex chromosome changes in the Syngnathidae species appear to have preserved male heterogamety, including in the two seahorse species studied here, which diverged about 11.4 Ma. This is consistent with the prediction that turnover events preserving the same heterogametic sex should be more frequent than those changing the heterogamety (Bull 1983). This has also been found in large comparative studies of true frogs (Jeffries, et al. 2018) and cichlids (Gammerdinger and Kocher 2018; El Taher, et al. 2021). It has been suggested that sex chromosome turnovers are responses to genetic degeneration and accumulated deleterious mutational load (Blaser, et al. 2013), to sexual conflict(van Doorn and Kirkpatrick 2007), or both together in the “hot potato” model of Blaser et al. (2014) (which predicts conservation of patterns of heterogamety and non-random recruitment of certain autosomes as sex chromosomes during their transition), or to meiotic drive (Úbeda, et al. 2015), or that they are selectively neutral changes under genetic drift (Veller, et al. 2017). The reasons for individual turnover events are unknown, but many species, including the two seahorse species studied here, do not have strongly degenerated sex-linked regions chromosomes as required by the hot potato model, which suggests that transitions can occur without extensive accumulated deleterious mutations.

A factor that is rarely mentioned is the possibility that the recombination landscape might pre-date a turnover. Ancestrally low recombination across large genomic regions, either in males or in both sexes, favors sex chromosome transitions. This is because, if a new male-determining factor arises within such a region, this immediately creates a region of complete (or almost complete) sex-linkage that is transmitted like a sex chromosome (Bergero, et al. 2019). The same may have occurred in turnover events in tilapias, which are members of the cichlid group of fish (Tao, et al. 2021).

Localization of crossovers to the ends of chromosomes in meiosis of males (or both sexes) may be widespread among other teleosts (Sardell and Kirkpatrick 2020), as well as in amphibians (Dufresnes, et al. 2021) and plants (Charlesworth 2019; Rifkin, et al. 2021). Accurate fine-scale sex-specific genetic maps are scarce for teleosts, apart from the guppy (Bergero, et al. 2019), some sticklebacks (Sardell, et al. 2018, 2021), and the Atlantic halibut (Edvardsen, et al. 2022) and Atlantic herring (Pettersson, et al. 2019), so only crossover localization in both sexes will often be detectable. Among these species, strong male-specific crossover localization was found in all except for the Atlantic herring. Our results in seahorse species suggest that recombination was indeed ancestrally low in the current Y-linked regions, though no sex-specific recombination map is available in *H. abdominalis* or *H. erectus*, and our genome-wide estimates provide only sex-averaged recombination rates. However, as mentioned above, microsatellite analyses in one seahorse species *H. angustus* (Jones, et al. 1998) found a much higher recombination rate in females than in males, and the GC spikes at the tip regions of chromosomes in the two seahorses species studied here (fig. 1) suggest crossover localization similar to that in the other fish species mentioned above. This should be further tested by genetic mapping in these species. Newly developed methods using linked-read sequencing of sperm DNA (Dréau, et al. 2019; Yoshitake, et al. 2022) hold promise for future examination of crossover patterns in seahorse species.

We also found that the present boundary between the PAR and putatively sex-linked region of HaCh6 coincides with the location of an ancient centric fusion between two chromosomes, which occurred long before this chromosome became a sex chromosome. After the fusion, this genome region must have been a centromeric or pericentromeric region, probably with a lower recombination rate than other parts of the new (fused) Chr6, even in females. If crossovers in this ancestral species were localized to the chromosome ends in males, the fusion could have created larger regions of low recombination on one or both arms. If the chromosome gained a male-determining factor in a turnover event, this would create a new Y-linked region.

### Non-randomness of Chromosomes Involved in Sex Chromosome Turnovers, and Subsequent Evolutionary Changes in New Sex-linked Regions

Sex chromosome turnovers can occur in two ways (Pan, et al. 2016; Vicoso 2019), either through translocation of a gene or gene duplication with a sex-determining function to a new genomic location (as in the house fly, see (Sharma, et al. 2017), or common seadragon and alligator pipefish (Qu, et al. 2021), or by acquisition of sex-determining function by a mutation in a pre-existing gene (termed “diversification”). In the former case, any chromosome might gain a new sex-determining factor, though this is most likely when a SA polymorphism is maintained in the new location (van Doorn and Kirkpatrick 2010). If so, new sex-determiners are expected to arise in genome regions already carrying genes involved in gonad functions, or other functions where SA mutations may arise. In the diversification process, chromosomes carrying genes involved in the sex-determining cascade are more likely than others to become sex chromosomes. Some chromosomes are indeed observed to gain sex-determining factors more often than others in amniotes (O'Meally, et al. 2012; Kratochvíl, et al. 2021) and even within the cichlids (Gammerdinger and Kocher 2018; El Taher, et al. 2021). The ancestral gene content could thus have facilitated the gain of a new male-determining gene.

Chr4 is enriched for testis-biased genes in both studied seahorse species (fig. 4*B*). It is also the XY sex chromosome in another Syngnathidae species, the common sea dragon (Qu, et al. 2021) (supplementary fig. S34, Supplementary Material online) due to a recent insertion creating the *AMHR2Y*; it is homologous to a part (21 Mb or 32.7%) of a Nile tilapia chromosome LG7 (supplementary fig. S35, Supplementary Material online) that has convergently evolved to become a sex chromosomes in multiple other cichlid species, in Lake Tanganyika (including in the families of Eretmodini, Bathybatini, Perissodini, and in the species *Hemibates stenosoma* (El Taher, et al. 2021); figure 2*D*). These results are consistent with the hypothesis that the ancestral chromosome carried genes capable of acquiring mutations conferring an upstream male-determining function, making it especially likely to become a sex chromosome (rather than that recent evolution of Y-linkage triggered changes toward higher expression in testis).

In contrast, after part of HaChr6 became sex-linked we detect increased pouch-specific transcription that was not seen in the autosomal orthologs in *H. erectus*. This may reflect the predicted “masculinization”, in which newly Y-linked alleles with male-related functions undergo adaptive evolution. In a seahorse species, any pouch genes in the new Y-linked region may be the first to adapt to such a genomic change. Pronounced genetic degeneration is probably expected to evolve later than such adaptive changes, as it depends on slower processes involving genetic drift of deleterious mutations, or their hitch-hiking effects along with advantageous mutations, under complete sex-linkage (Bachtrog 2008). Consistent with this, the evidence presented here shows that the seahorse Y-linked regions have not lost high proportions of genes, despite being large genome regions. They may have evolved recently, or degeneration of these regions may have been slowed by occasional recombination with the X, as is suggested by the small Y–X divergence estimates for both HaChr6 and HeChr4.

## Materials and Methods

### DNA Sampling and Sequencing

The big-belly seahorse and lined seahorse samples used in this project were obtained from cultured populations in Fujian (China), with approval by the Animal Ethical and Welfare Committee of the Fisheries Research Institute of Fujian (Approval No. IACUC-2019-031). The original population of the big-belly seahorse was established from about 10,000 individuals caught from the wild, and that of lined seahorse from about 2,000 to 3,000 individuals. Before tissue collection, all the seahorses were cultured in indoor breeding tanks (150L), with seawater from the sea and adjusted to a salinity of 31 ‰ and temperature 16–18°C. The seahorses were fed three times per day (08:00, 12:00, and 16:00) with mysid. All the seahorses were sacrificed after being anesthetized for 30 min by MS-222 solution at a concentration of 50 mg/L, which is approved by FDA (Food and Drug Administration, USA). To infer the sex-linked regions, we collected 30 male and 29 female individuals of *H. abdominalis*, and 10 male and 10 female *H. erectus*. Muscle tissue from each individual was dissected under a stereomicroscope and stored at −80°C until processing. DNA extraction was carried out using EasyPure® Genomic DNA Kit (TransGen Biotech, China). A paired-end library was constructed with an insert size of 250 base pairs (bp) according to the protocol provided by the manufacturer, and was sequenced on an Illumina X ten platform by Annoroad Gene Technology Co. Ltd. (http://www.annoroad.com).

### mRNA Sequencing and Gene Expression Analysis

We collected tissues from brain, kidney, testis, ovary, male brood pouch and female abdominal skin of *H. abdominalis* individuals, at both immature (3 months old) and mature (5 months old) stages. For each tissue sample, we prepared two biological replicates for total RNA extractions using the EasyPure RNA Kit (TransGen Biotech, China) following the manufacturer's protocol. Unstranded total RNA library was constructed and sequenced with a read length of 150 bp using the Illumina HiSeq 2000 sequencing platform. The recently assembled *H. abdominalis* genome (He, et al. 2021), as well as the published genomes of *H. erectus, S. scovelli,* and *Oreochromis niloticus* (Small, et al. 2016; Lin, et al. 2017; Tao, et al. 2021) were used as references for gene expression analyses. A chromosome-level genome assembly of *H. erectus* was based on the *H. abdominalis* reference genome (Alonge, et al. 2019) using RaGOO. To investigate biased gene expression in different tissues in *H. erectus*, we also collected published transcriptome data from *H. erectus* testis, ovary, brood pouch, muscle, and kidney (Lin, et al. 2016). RNA-seq reads were mapped to the respective genomes using Hisat2 (Kim, et al. 2015) (2.1.0) and the reads mapped to each gene were counted using featureCounts version 1.6.2 (Liao, et al. 2014). Read counts were normalized using the TPM method. Differentially expressed gene analyses to compare tissue types, developmental stages and sexes were performed with the edgeR R packge (Robinson, et al. 2010). Genes with low expression were filtered with the filterByExpr function in the edgeR package, using the default parameters (min.count = 10, min.total.count = 15, min.prop = 0.7). We used the Benjamini and Hochberg's algorithm (BH) to control the false discovery rate (FDR). We classified only genes with BH-adjusted *P*-value <0.05 (exact negative binomial test) as significantly differentially expressed. After excluding genes not expressed (TPM < 1) in any tissue, we estimated each gene's tissue-specificity according to the tau (τ) algorithm(Yanai, et al. 2005), which performed best in recognizing tissue-specific genes in a benchmark study (Kryuchkova-Mostacci and Robinson-Rechavi 2017). To enable comparisons across different tissues, we performed a quantile normalization on the entire dataset before calculating tau values using the tispec R-package (https://rdrr.io/github/roonysgalbi/tispec). The threshold for a gene to be classified as tissue-specific was set to 0.8, following a previous study (Kryuchkova-Mostacci and Robinson-Rechavi 2017). If a tissue-specific gene defined by τ value also had a top-three normalized expression level in a tissue, we classified it as expressed specifically in that tissue. We also did analyses after defining tissue-biased genes as genes with at least 2-fold greater TPM than the mean TPM in other tissues of the same developmental stage, to test whether the conclusions our robust to different definitions.

### Annotation of Repetitive Sequences

We used RepeatModeler version 2.0 (Flynn, et al. 2020) for de novo identification of repetitive elements in the genome sequences of the species studied here, and to classify them based on their sequence similarity to known repeat families from other organisms. We then combined the all repeat families identified with annotated repeats in the Dfam_3.1 (Hubley, et al. 2016) and RepBase-20170127 databases and used RepeatMasker version 4.0.9 (Tarailo-Graovac and Chen 2009) to annotate the repeat sequences in our genome sequences.

### Comparative Genomics Analysis

Chromosome-level genome assemblies of teleost species, the Gulf pipefish, *S. scovelli* (Small, et al. 2016), *Nile tilapia*, *O. niloticus* (Tao, et al. 2021) and Guppy, *Poecilia reticulata* (Kunstner, et al. 2016), as well as the genome of spotted gar, *L. oculatus* (Braasch, et al. 2016) were aligned to the chromosome-level assembly of the big-belly seahorse, *Hippocampus abdominalis* (He, et al. 2021), using a large-scale alignment tool, LAST (https://gitlab.com/mcfrith/last), using the commands: lastdb -uNEAR -R01 ./out.ref ref.genome.fa; lastal -P32 ./out.ref query.genome.fa | last-split >./out.maf; maf-swap./out.maf | last-split >./out.1to1.reverse.maf; maf-swap./out.1to1.reverse.maf >./out.1to1.maf. Syntenic relationships were visualized with the Circos software, version 0.69.9 (Krzywinski, et al. 2009).

### Identification of the Candidate Sex–Determining Regions

To attempt to identify the sex-linked region of *Syngnathus scovelli* (the Gulf pipefish), demultiplexed ddRAD-seq data (Flanagan and Jones 2017) from males and females (accession #: PRJNA358088) were downloaded from NCBI Sequence Read Archive (SRA). Demultiplexed reads were analyzed with the RADSex computational workflow (Feron, et al. 2021) using the *radsex* software version 1.1.0 (https://github.com/SexGenomicsToolkit/radsex). Tables of sex-associated markers and their depths of coverage were created with *process* command. The distribution of markers in the two sexes was computed with *distrib,* and markers significantly associated with phenotypic sex were extracted with *signif* using variants with a minimum depth of 5 (–d 5) for both commands, and the default for all other settings. 611,484 markers with minimum depth = 5 were found in at least one individual, among which 419 were significantly associated with sex (*radsex signif*, *P* < 0.05, χ^2^ test with Bonferroni correction, highlighted tiles in supplementary fig. S15, Supplementary Material online). Among these 419 markers, 53 (12.6%) had reads aligned to unanchored scaffolds, and 263 (62.8%) had reads not uniquely aligned with mapping quality higher than 15. The remaining 103 markers were distributed sparsely among the 22 LGs, with between 1 and 15 markers on each LG. F_ST_ between the Gulf pipefish male and female populations was computed with the –fstat option in the Stacks software, version 2.5.3 (Rochette, et al. 2019).

We performed whole genome resequencing of 30 male and 29 female individuals to infer the sex-linked region in *H. abdominalis*, plus 10 male and 10 female individuals of *H. erectus*. For each individual, sequencing reads were mapped to the reference genome with BWA-mem (Li and Durbin 2009) (0.7.17-r1188). We called the SNPs using HaplotypeCaller in GATK (DePristo, et al. 2011) (3.8.1.0) and then called the genotypes with GenotypeGVCFs. The SNP results were filtered with following filtering expression: “QD < 2.0 || FS > 60.0 || MQRankSum < −12.5 || RedPosRankSum < −8.0 || SOR > 3.0 || MQ < 40.0”. Only biallelic sites with minor allele frequency greater than 0.05 were kept. A total of 6,115,914 and 5,603,399 SNPs in *H. abdominalis* and *H. erectus*, respectively, passed the quality control. We used Beagle version 5.0 (Browning, et al. 2018) to impute missing genotypes, and SHAPIT v2.r904 (Delaneau, et al. 2013) for haplotype estimation (phasing). A GWAS was performed by mixed-model association using eXpedited (beta-07Mar2010 version of EMMAX); (Kang, et al. 2010), using sex as a phenotype. Phased genotypes were processed with PLINK v1.90b6.10 (Purcell, et al. 2007); http://pngu.mgh.harvard.edu/purcell/plink/) to generate the input for EMMAX. The threshold for genome-wide significance in the GWAS was set at *P*-value = 8.18e-9 for *H. abdominalis*, and 8.92e-9 for *H. erectus* by dividing 0.05 with the number of total SNPs in each species. For *H. abdominalis*, the sex-linked region was inferred based on presence of SNPs significantly associated with sex on HeChr6 by the GWAS analysis. Phasing either with or without imputation did not influence the enrichment of sex-associated SNPs in the sex-linked region of *H. abdominlais* Chr6 or *H. erectus* Chr4. The boundaries of the HeChr4 region were inferred on the same basis and using the density of sites at which males are heterozygous and females are homozygous. We used nucmer (Marçais, et al. 2018) to infer the homologous regions of identified seahorse sex-linked regions in other species. Only one-to-one homologous alignments were kept. We validated the genotypes of 10 randomly picked *H. abdominalis* sex-associated SNP sites by performing polymerase chain reaction (PCR) experiments using FastLong PCR MasterMix (PC8001, Aidlab) according to the manufacturer's protocol, followed by sequencing to identify the SNP genotypes. The primers used are listed in supplementary Table S8, Supplementary Material online.

We used 50 kb overlapping sliding windows with step size 25 kb to calculate the F_ST_ values between male and female populations, using vcftools version 0.1.13 (Danecek, et al. 2011), and coverage depths of merged reads from males or females, using sambamba version 0.7.0 (Tarasov, et al. 2015). Heterozygous sites found only in males were defined after excluding the possibly sex-reversed individuals (see the Results section). Synonymous site divergence between X and Y was calculated based on such male-specific SNPs using the KaKs_Calculator software (Zhang, et al. 2006) and Nei and Gojobori's (Nei and Gojobori 1986) correction for multiple changes. F_IS_ was calcaulated according to Nei (Nei 1973), F_IS_ = 1–*H*_o_/*H*_e_, where *H*_o_ and *H*_e_ represent observed and expected heterozygosities of each SNP.

Recombination rates (rho) were estimated after excluding SNPs with >10% missing individuals in our sample, or located within repetitive regions, using FastEPRR2.0 (Gao, et al. 2016) with parameters winLength = 50,000, stepLength = 25,000, winDXThreshold = 10. Change-point analyses of GC%, F_ST_, the density of male-specific heterozygous sites, and log-transformed *P*-values in our GWAS, were conducted with the R-package “changepoint” (Killick and Eckley 2014) to implement the binary segmentation method (Bai 1997), with a maximum of three changepoints; changepoints were detected based on both mean value and variance.

### Searches for Inversions in the *H. abdominalis* and *H. erectus* Genomes

We searched for read pairs supporting inversions in our candidate sex-linked region based on their orientation and mapping coordinates, using TIDDIT (Eisfeldt, et al. 2017) (https://github.com/SciLifeLab/TIDDIT). Read pairs aligning on the same chromosome at a distance higher than the insert size, and having the same orientation, were considered as candidates supporting inversions. The fractions of read pairs supporting inversion events were used to define the genotypes in these regions, defining fractions less than 100% and greater than 25% as candidate heterozygotes, and less than 25% as homozygotes for the reference arrangement. Inversion events were identified in PacBio sequences using npINV (Shao, et al. 2018). PacBio reads containing a pair of alignments mapping to the same chromosome, but with a different orientation, were considered to support inversions.

### A/B Compartments

We processed the Hi-C data (He, et al. 2021) with HiCPro (Servant, et al. 2015). Mapped read pairs were used to generate raw Hi-C contact matrix at a 50 kb resolution using hicBuildMatrix of the HiCExplorer (2.2.1) suite (Wolff, et al. 2018, 2020). We used the ICE method implemented in hicCorrectMatrix to remove the bins with zero or low number and extremely high number of reads because the former bins usually tend to indicate repetitive regions and the latter bins may represent copy number variations. After correcting the Hi-C matrices (hicCorrectMatrix), we used cooltools (0.3.2) (https://cooltools.readthedocs.io/) and its call-compartments function to obtain the first eigenvector values (PC1) of each chromosome of the Hi-C matrices with a 50 kb resolution. Regions with positive PC1 values were assigned as A (active) compartments and those with negative PC1 values were assigned as B (inactive) compartments, adjusted by the GC percentage of the region.

### GO and Phenotype Enrichment Analysis

Orthologs of *H. abdominalis* and *H. erectus* found in the *Danio rerio* (zebrafish) genome assembly GRCz11 (danRer11) and *Mus musculus* (house mouse) genome assembly GRCm39 (mm39) from NCBI (National Center for Biotechnology Information) were used as input in Gene Ontology and phenotype enrichment analysis. We performed the GO analyses by Metascape (Zhou, et al. 2019) with default parameters (*P* < 0.01, min overlap = 3 and min enrichment = 1.5). We used phenome2 (Weng and Liao 2017) (http://evol.nhri.org.tw/phenome2/) to detect the enriched phenotypes in each gene set. Zebrafish and mouse phenotypes with Bonferroni corrected *P*-value < 0.01 (Fisher's Exact test) were considered as enriched.

## Supplementary Material

msac279_Supplementary_Data

## Data Availability

Whole genome sequencing reads from both sexes of *Hippocampus abdominalis* and *Hippocampus erectus* individuals, as well as RNA-seq reads of *H. abdominalis* tissues have been deposited on NCBI Short Reads Archive under the BioProject Accession Number PRJNA736169. Other published data used in this project are listed in supplementary Table S11, Supplementary Material online.
