# Supplementary material for: Independent Evolution of Sex Chromosomes and Male Pregnancy–Related Genes in Two Seahorse Species
Source: Mol Biol Evol. 2022 Dec 29;40(1):msac279. doi: 10.1093/molbev/msac279 (PMC9851323; doi:10.1093/molbev/msac279)
Supplement: msac279_Supplementary_Data [file msac279_supplementary_data.zip › Supplementary_Figures.docx]

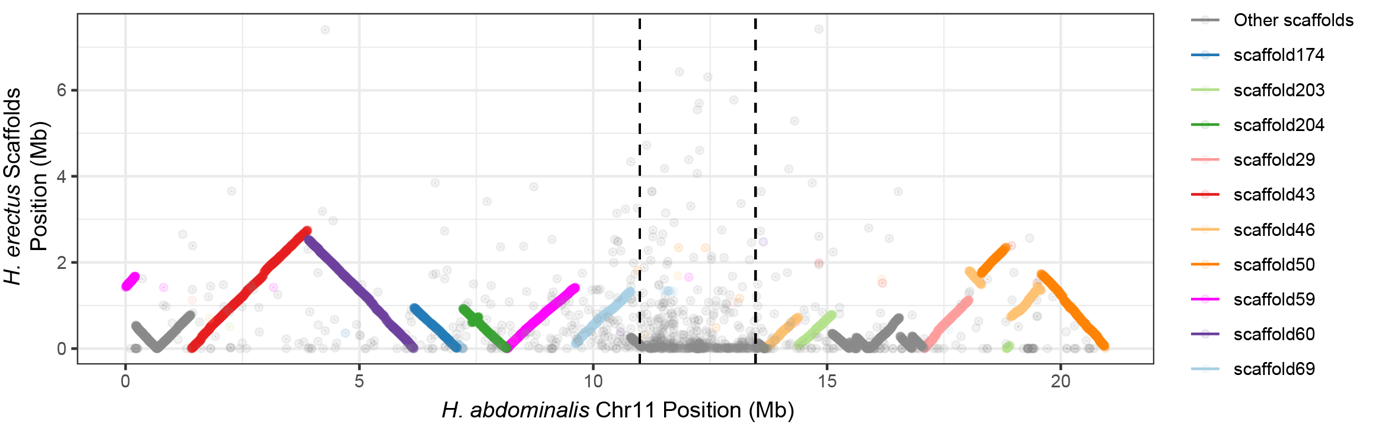


**Supplementary Figure S1. Genome alignment between *H. abdominalis* Chr11 and *H. erectus* scaffolds**

Dashed lines label the boundaries of the candidate centromeric region of *H. abdominalis* Chr11. None of the *H. erectus* scaffolds (indicated by different colors) exhibits continuous alignment spanning this region, indicating that Chr11 corresponds to two homologous chromosomes in *H. erectus*, suggesting a chromosome fusion led to formation of Chr11.


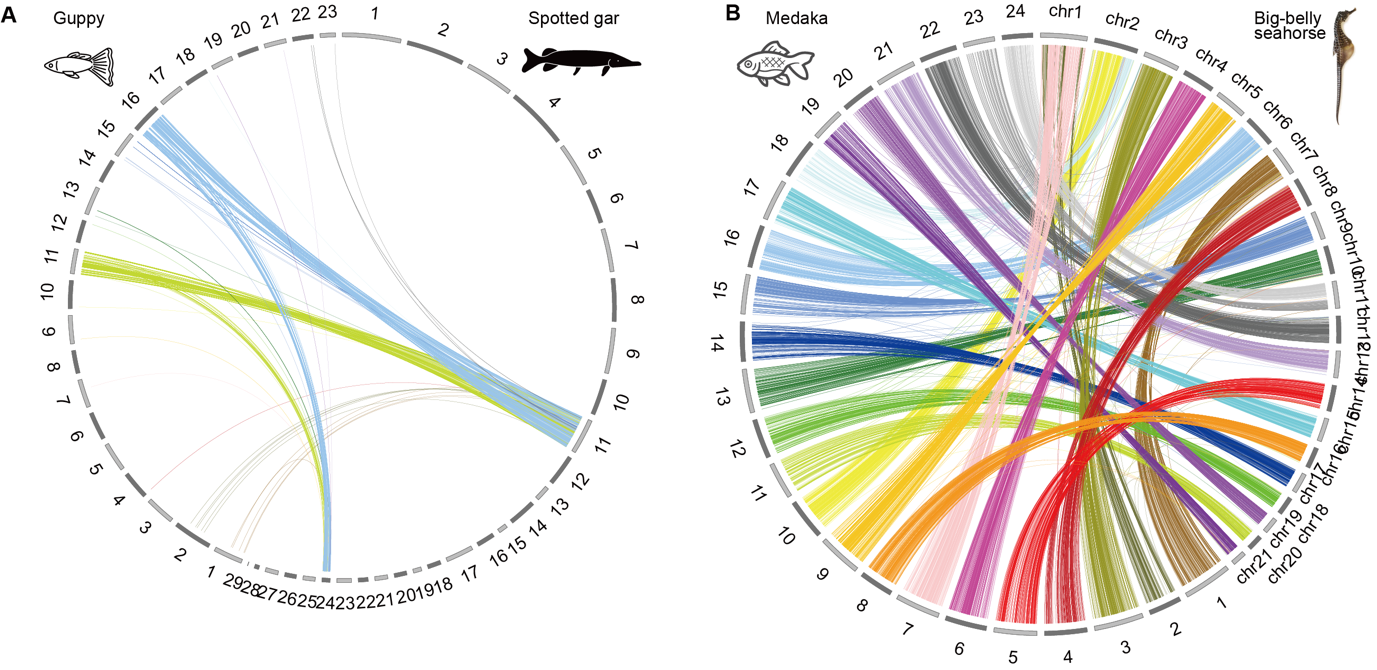


**Supplementary Figure S2. Two gar chromosomes are fused in Guppy and medaka.**

(A) Chromosome synteny between *Poecilia reticulata* (Guppy) and LG24 and LG11 of *Lepisosteus oculatus* (Spotted gar)*.* Each line represents an aligned sequence between LG24 and LG11 of the spotted gar and the guppy genome. Two distinct parts of the guppy LG16 are each homologous to one of these gar chromosomes, and the whole of LG16 is homologous to Chr6 of *H. abdominalis*. (B) Chromosome synteny between *Hippocampus abdominalis* (Big-belly seahorse) and *Oryzias latipes* (Medaka). Each line represents an aligned sequence between these two genomes. Chr6 of *H. abdominalis* also has one-to-one homologous relationship with LG16 of Medaka, indicating LG24 and LG11 of *Lepisosteus oculatus* is also fused in Medaka.


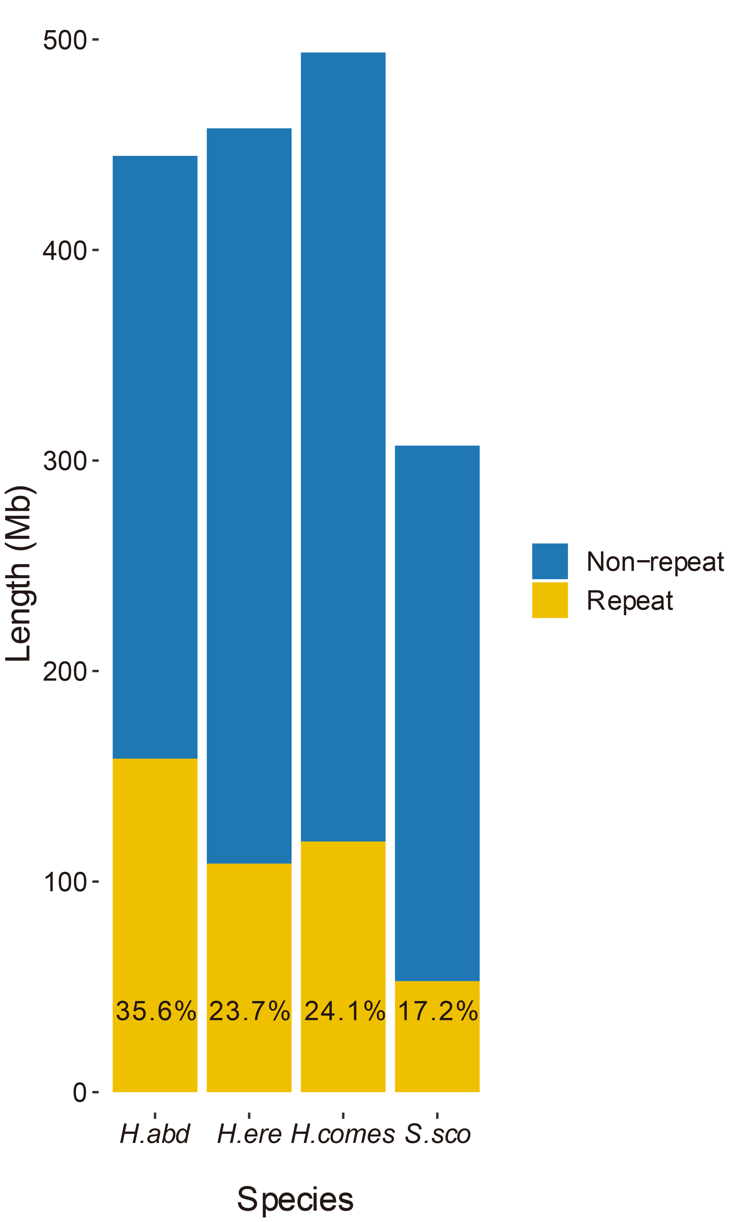


**Supplementary Figure S3. Genome size difference between seahorse and pipefish species.**

The plot shows the genome sizes and the contributions estimated to be made up of repeats in three seahorse species, *H. abdominalis*, *H. erectus*, *H. comes* and a pipefish, *S. scovelli*. *H. abdominalis* has a larger repeat content than the other species.


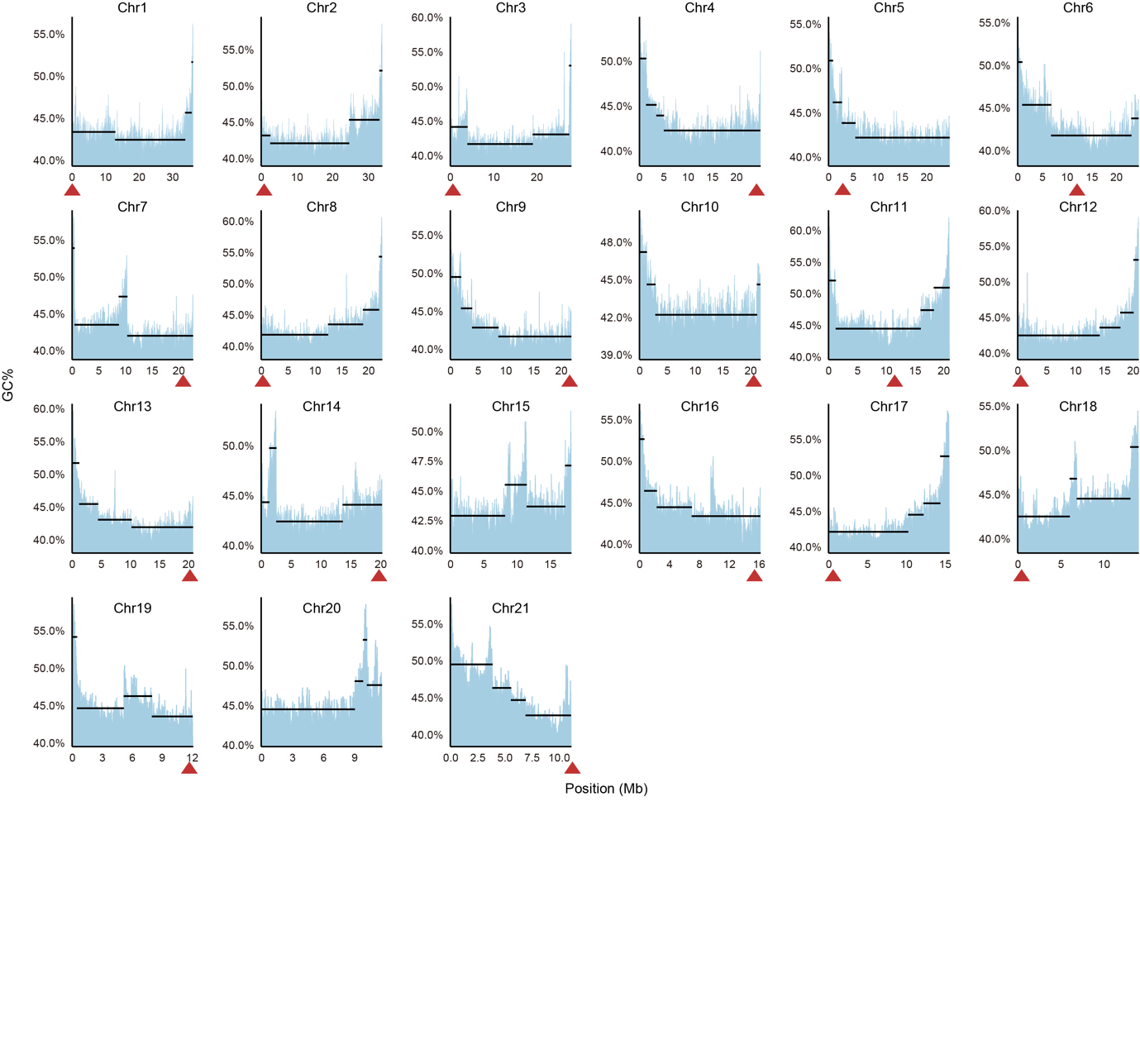


**Supplementary Figure S4. Change-point analysis of GC content of *H. abdominalis***

Each horizontal line represents the mean GC% of a segment identified by change-point analysis(Killick and Eckley 2014). Segments with the highest GC% on each chromosome, which account for 4.93% of the entire genome, are generally found in tip regions of the chromosomes. Red triangles labelled the position of candidate centromeric markers. Chr15 and Chr20 lack the centromeric markers.


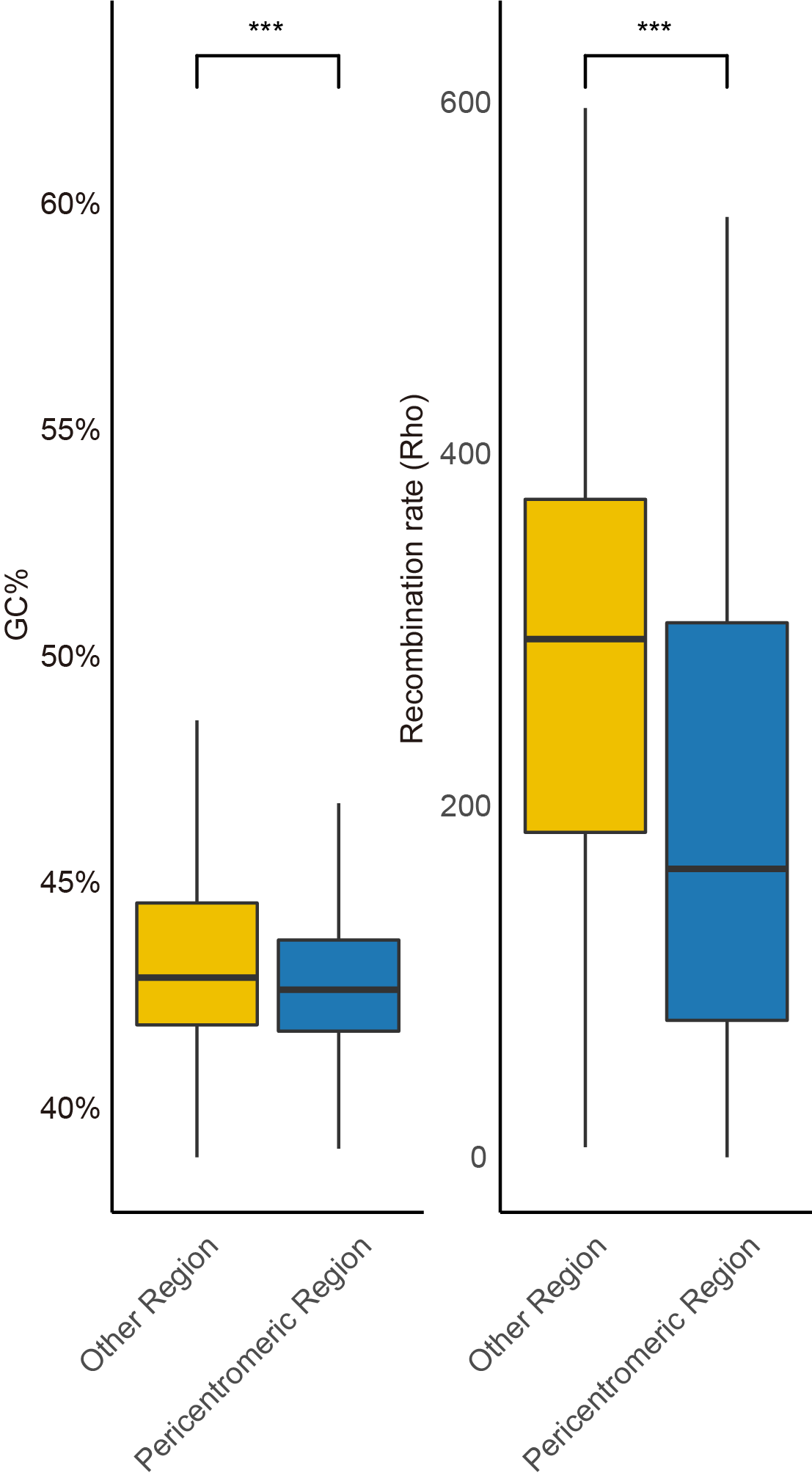


**Supplementary Figure S5. Comparison of recombination rate estimates (Rho estimates) and GC percentage between inferred pericentromeric regions and other regions of *H. abdominalis***

Recombination rate (Rho) and GC percentage estimates were calculated in each 50kb-sliding windows, separately for pericentromeric regions defined as the 3Mb regions flanking the putative centromeric marks **,** and other genome regions. The inferred pericentromeric regions have significantly lower GC content and sex-averaged recombination rate than other regions (P<0.001, Wilcoxon test).

**Supplementary Figure S6. GC content of *S. scovelli* linkage groups**

GC percentages were calculated in 50kb-sliding windows for each *S. scovelli* linkage group. Only 14 of the 22 linkage groups have patterns with GC spikes (GC% >55% at the end of linkage groups, p < 2.2e-16, Mann–Whitney U test) at one or both ends of linkage groups (indicated with red squares). The patter is therefore less clear than in *H. abdominalis*.


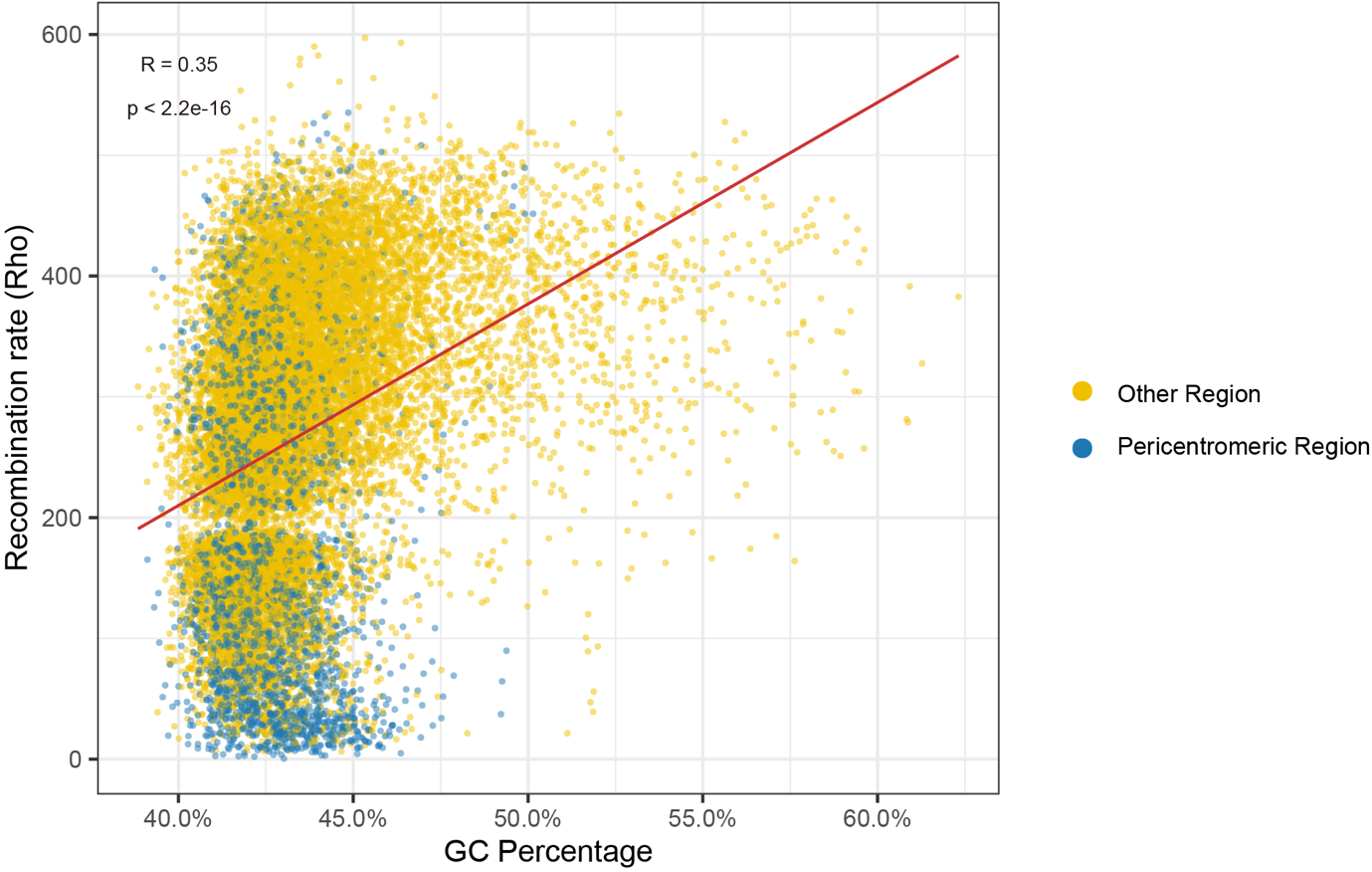


**Supplementary Figure S7. Correlation between the estimated recombination rate (Rho value, based on estimated linkage disequilibrium) and GC percentage in *H. abdominalis***

There is a significant positive correlation between recombination rate (Rho) and GC percentage in *H. abdominalis* (Pearson’s r=0.22, p<2.2e-16). The red line shows the linear regression. Windows represent pericentromeric regions and other regions were colored in blue and yellow separately.


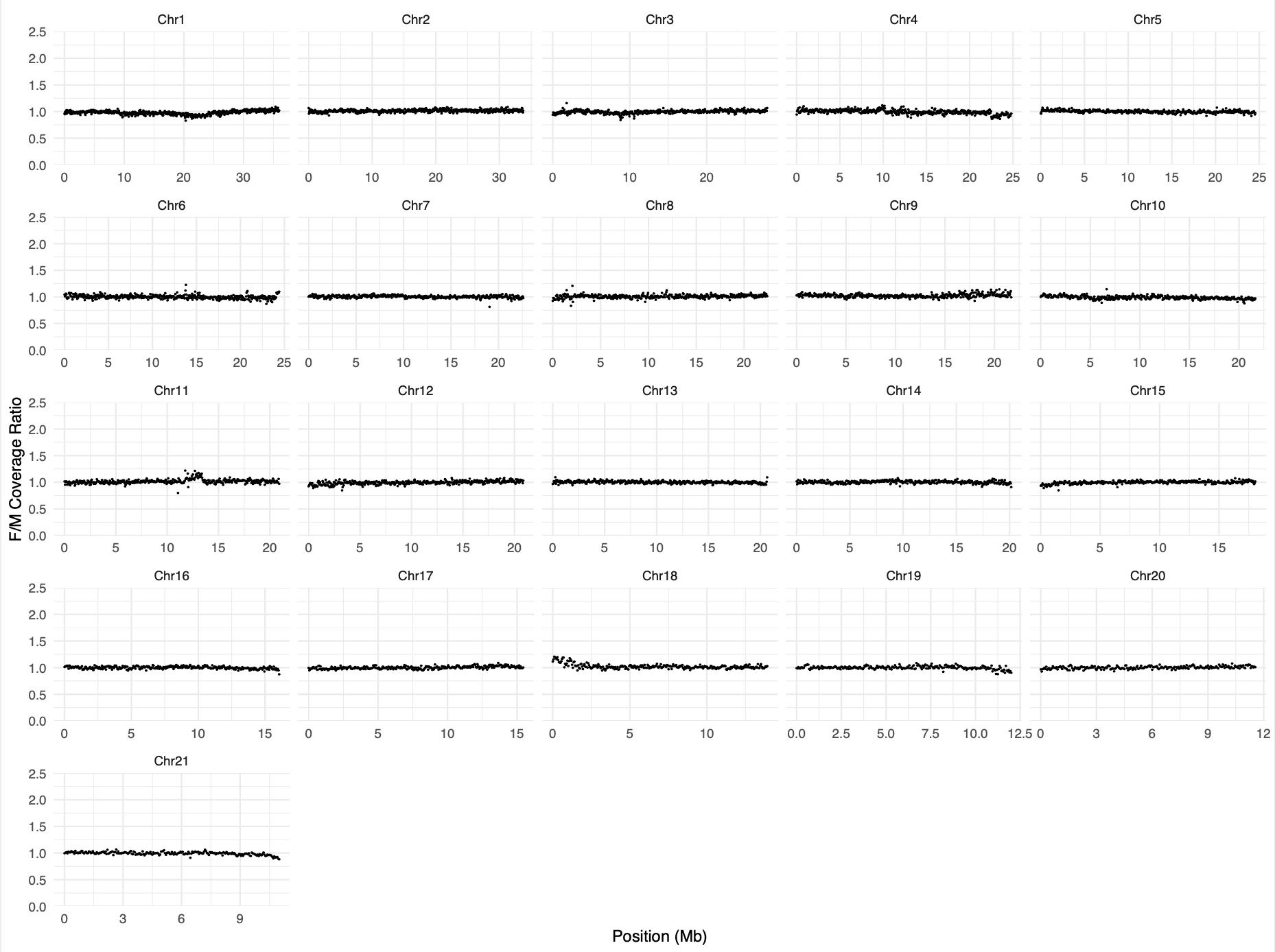


**Supplementary Figure S8. Coverage ratio between female and male samples of *H. abdominalis***

Coverage ratio between female and male samples of *H. abdominalis* were calculated in overlapping 50Kb-sliding window with 25kb steps. Only reads with MAPQ>20 were used. The only prominent coverage difference between the sexes is at the fusion site on Chr11.

**
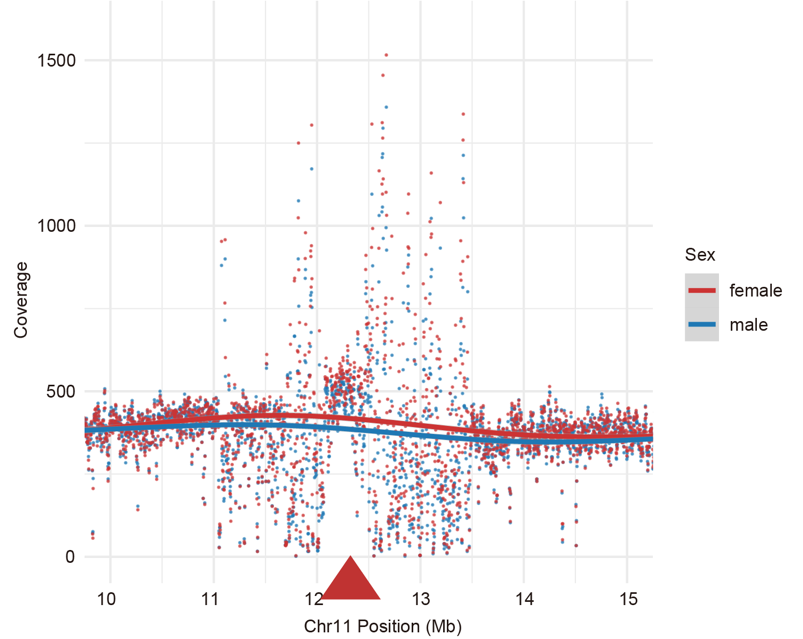
**

**Supplementary Figure S9. Coverage of female and male samples on Chr11 of *H. abdominalis***

Read coverage of female and male of *H. abdominalis* calculated in 5kb-sliding windows and normalized based on total read counts for each individual. Only reads with MAPQ>20 were used. The read coverage at the region are either very high or very low in the region surrounding the fusion breakpoint (indicated by a red triangle), compared to other chr11 regions. This may reflect this region is ancestrally centromeric and difficulties of accurate reads mapping due to presence of polymorphic repetitive elements in this region. Loess regression was used to fit a smooth curve to the points in the scatterplot.

**
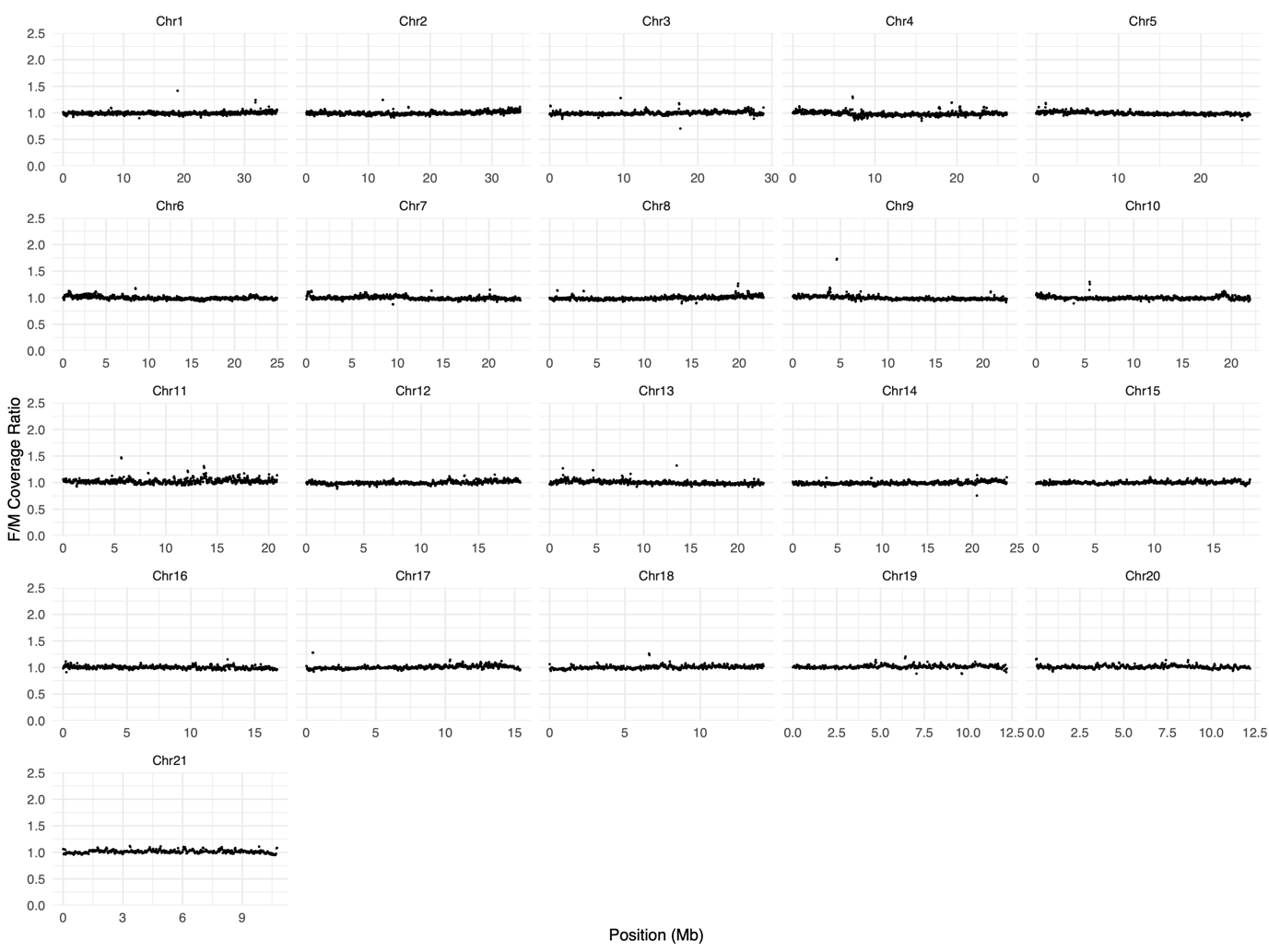
**

**Supplementary Figure S10. Coverage ratio between female and male samples of *H. erectus.***

Coverage ratio between female and male samples of *H. erectus* in overlapping 50Kb-sliding windows with 25 kb steps. Only reads with MAPQ>20 were used. None of the chromosomes exhibits prominent coverage difference between females and males.


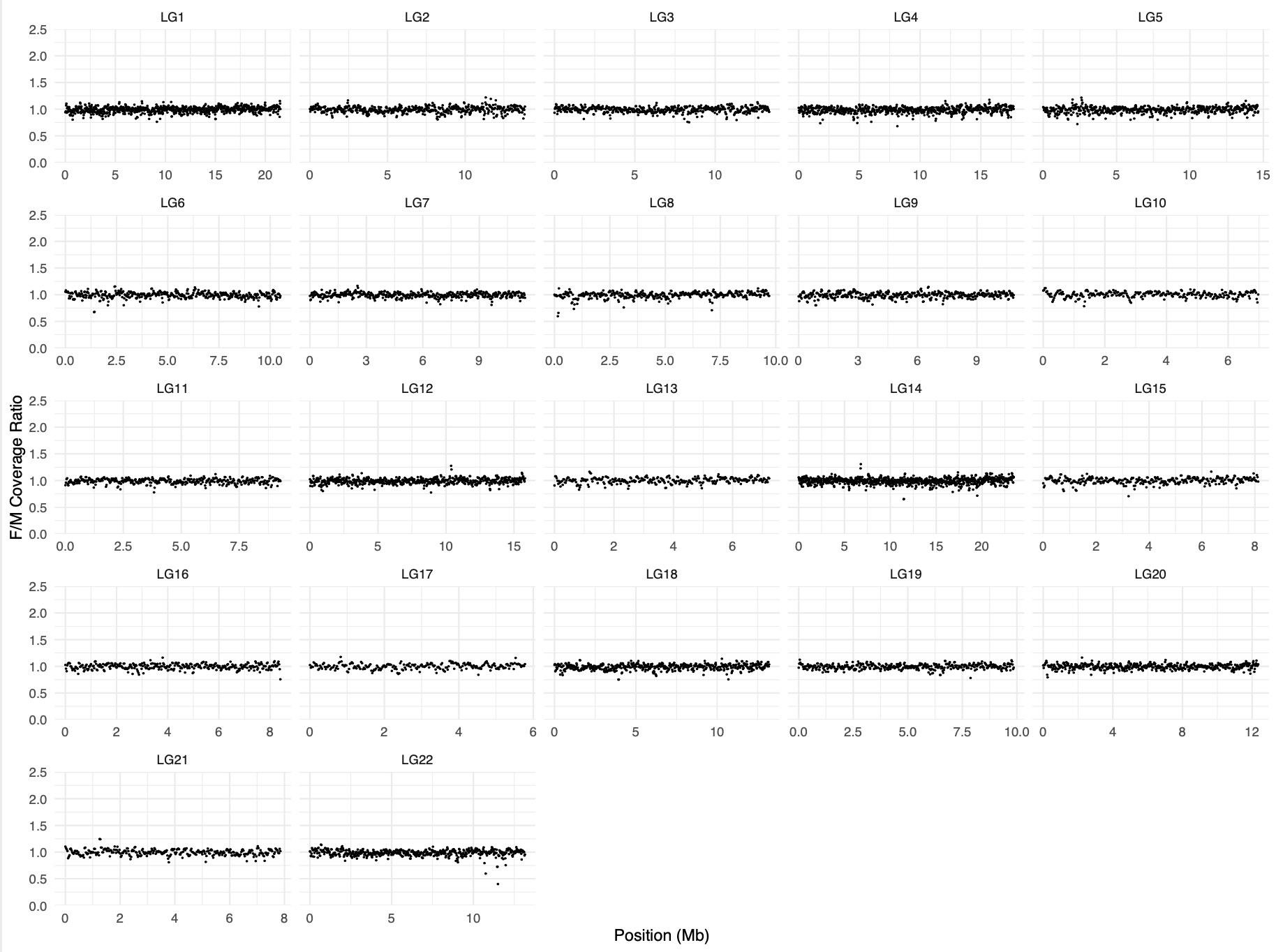


**Supplementary Figure S11. Coverage ratio between female and male samples of *S. scovelli.***

Coverage ratio between female and male samples of *H. erectus* in overlapping 50Kb-sliding windows with 25 kb steps. Only reads with MAPQ>20 were used. None of the chromosomes exhibits prominent coverage difference between females and males.


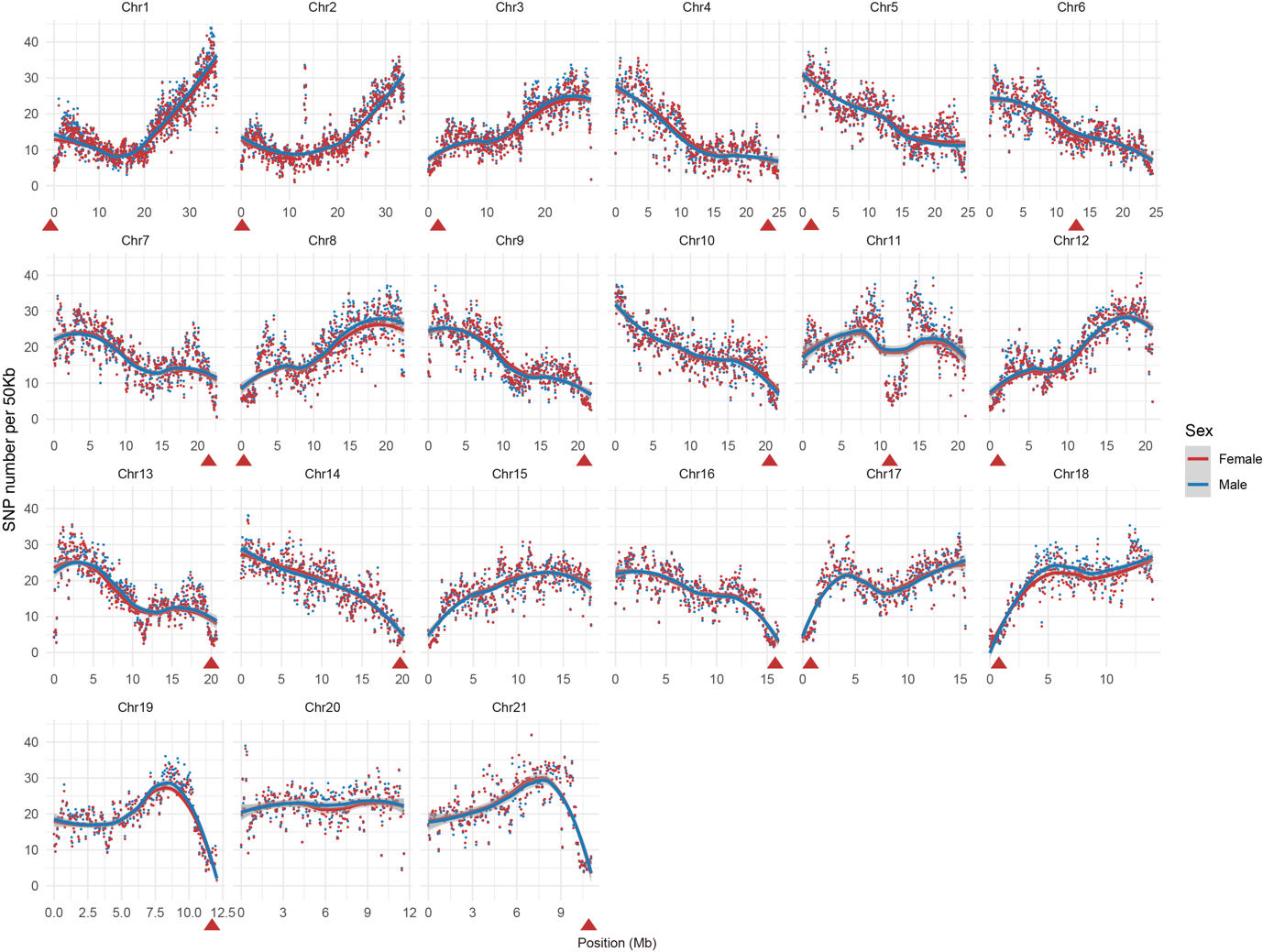


**Supplementary Figure S12. SNP density of female and male populations in *H. abdominalis***

SNP density of female (red) and male (blue) populations in *H. abdominalis* in 50kb windows. None of the chromosomes exhibits prominent SNP density difference between the female and male populations. The SNP density drops dramatically in the low GC regions of chromosomes. Loess regressions were used to fit a smooth curve (blue for male and red for female) for the points in the scatterplot. Red triangles labelled the position of candidate centromeric markers.


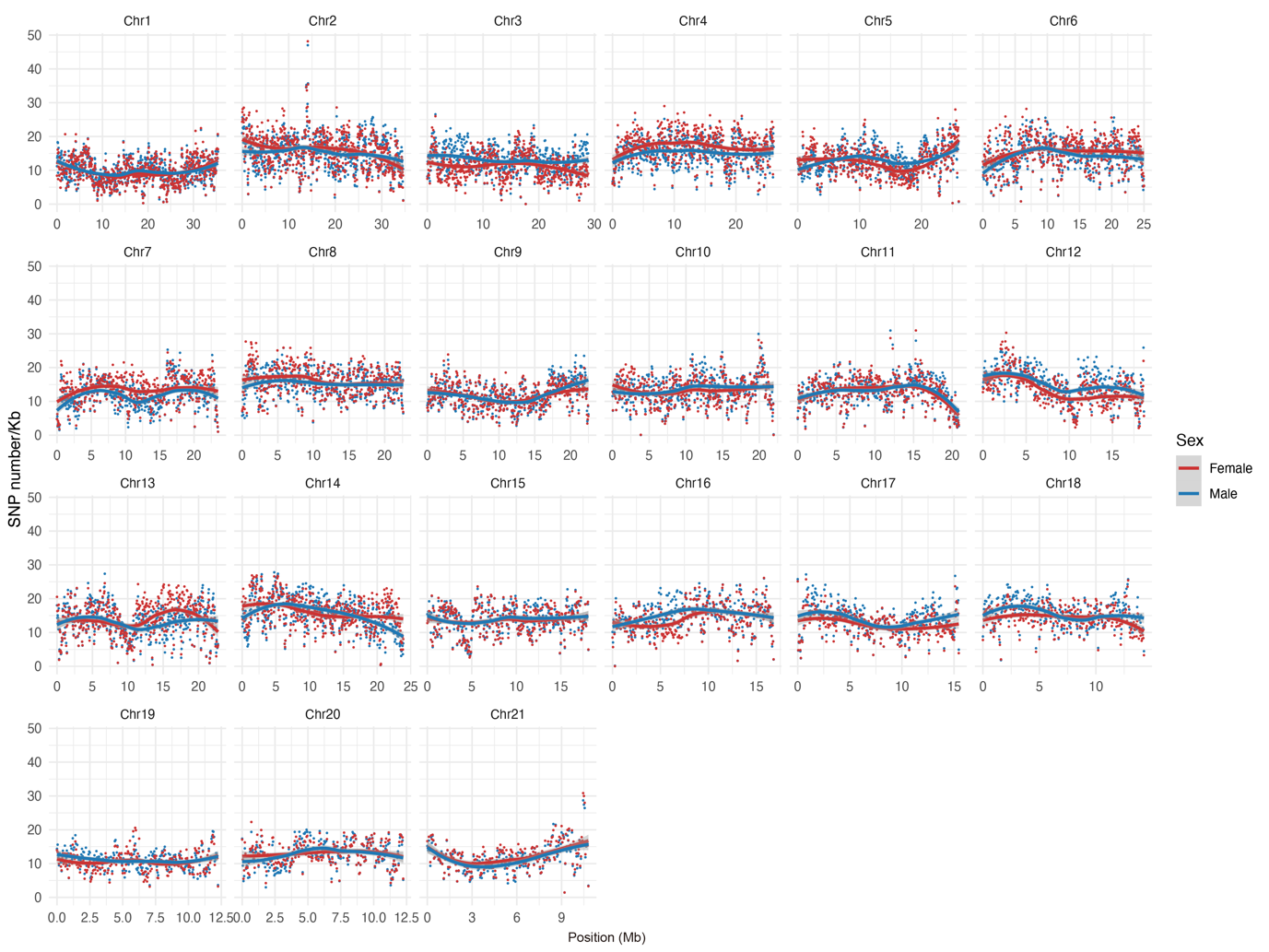


**Supplementary Figure S13. SNP density of female and male populations in *H. erectus***

SNP density of female (red) and male (blue) populations in 50kb windows, with Loess regressions used to fit smooth curves (blue) as for *H. abdominalis*. In *H. erectus*, the SNP density does not differ greatly in relation to the GC content. This may also reflect the low SNP density region at chromosome ends in H. erectus genome was not well-assembled.


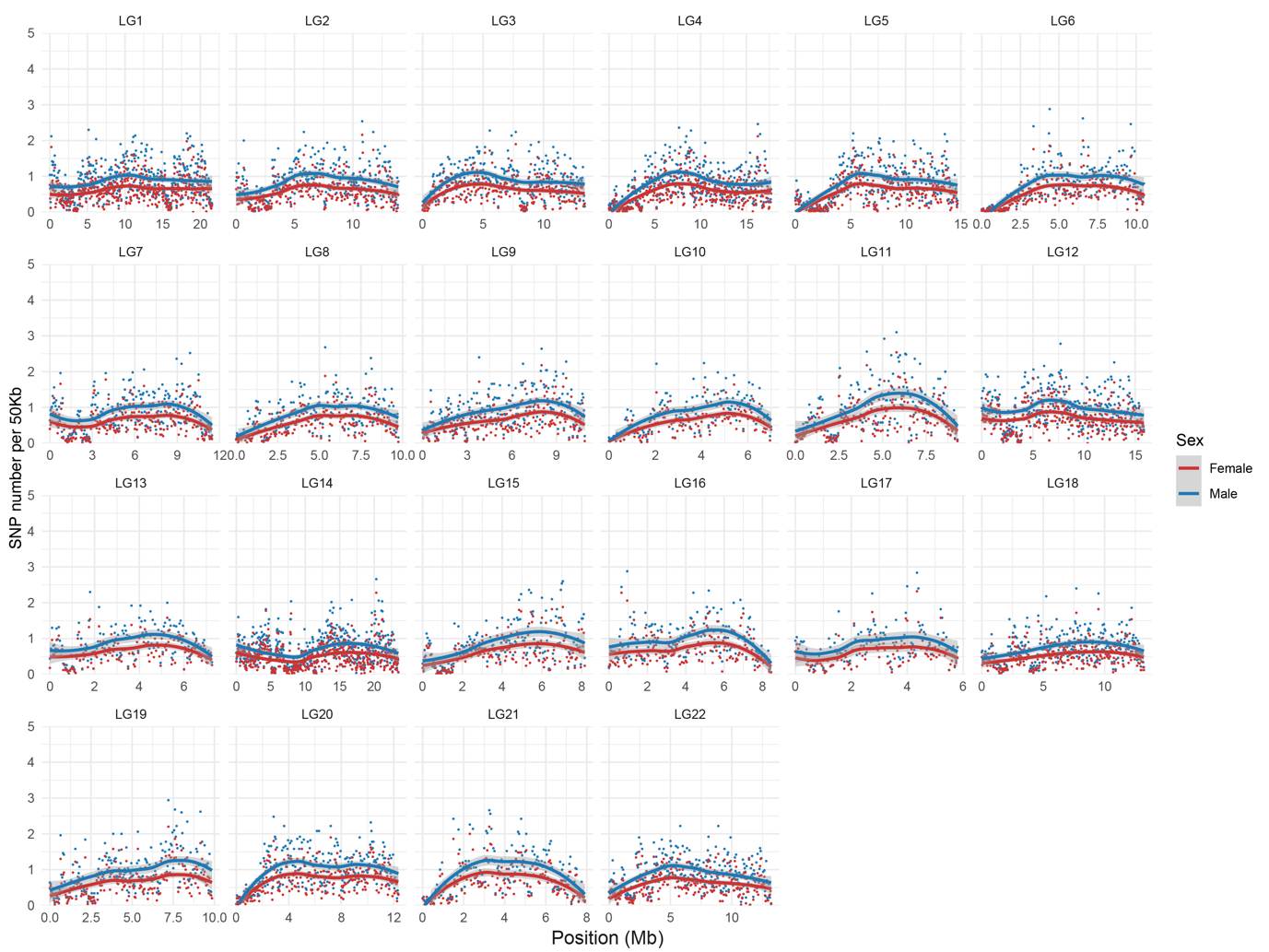


**Supplementary Figure S14. SNP density of female and male populations in *S. scovelli***

SNP density of female (red) and male (blue) populations in 50kb windows, based on RADseq data, with Loess regressions used to fit smooth curves (blue) as for *H. abdominalis*. Since RADseq has a limited resolution, the SNP density in both sexes is only about 1 SNP per 50Kb, much lower than in the species shown in the previous two figures.


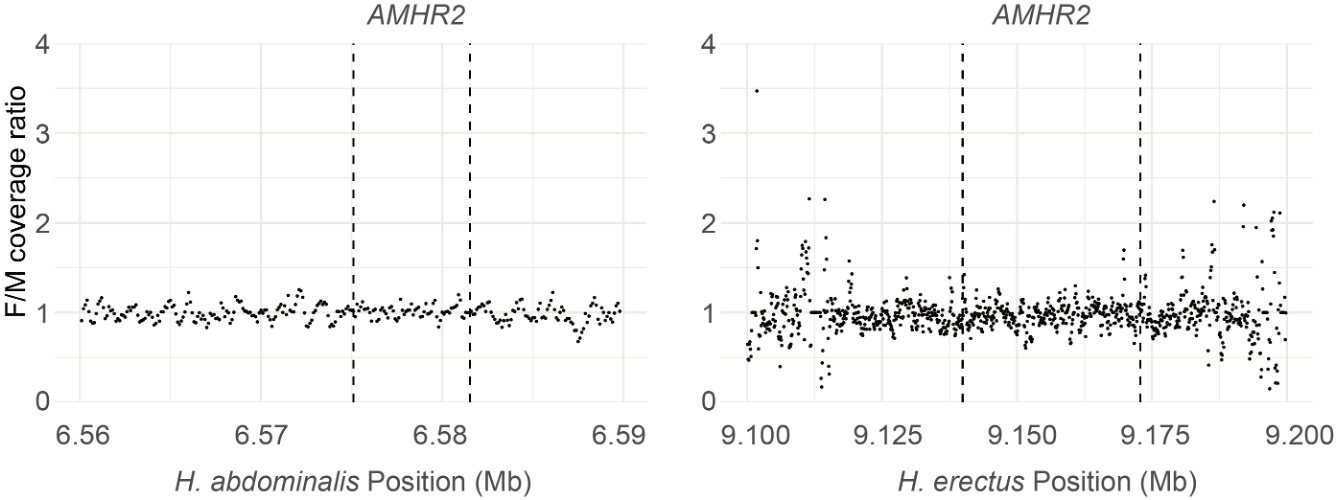


**Supplementary Figure S15. Coverage ratio between males and females in the *AMHR2* gene region of *H. abdominalis* and *H. erectus.***

Coverage ratio between female and male samples of *H. abdominalis* and *H. erectus* in 100bp non-overlapping windows, showing the similar coverage in both sexes in the region.


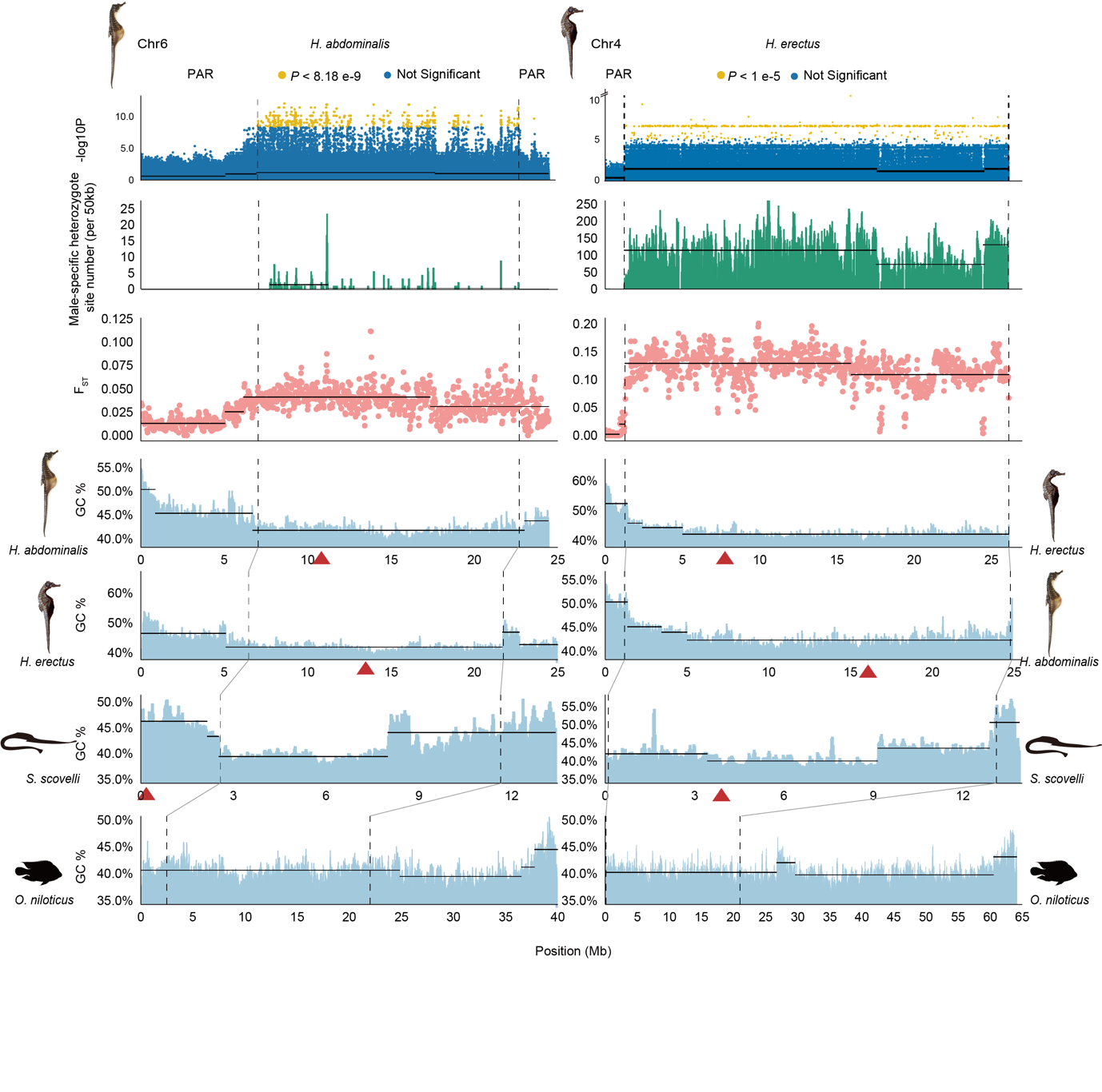


**Supplementary Figure S16. GC content in *H. abdominalis* Chr6 and *H. erectus* Chr4.**

The boundary between the sex-linked region and PAR was identified from coinciding sharp transitions in the P-value of GWAS, and of heterozygotes in males, and also higher F_ST_ values between the sexes, and a change GC content. Dashed vertical lines labelled the boundary between sex-linked region and PAR. Horizontal lines represent the mean value for each segment detected by change point analysis conducted by R package "changepoint" (Killick and Eckley 2014). Some transitions were not sharp in *H. abdominalis* but we inferred the PAR boundary at the left using the SNPs that were significant after Bonferroni correction and F_ST_ values. The putative sex-linked region has low GC in all Syngnathidae species, but not in the homologous region in the Nile tilapia genome. Red triangles are positions of putative centromeric markers in Syngnathidae species.


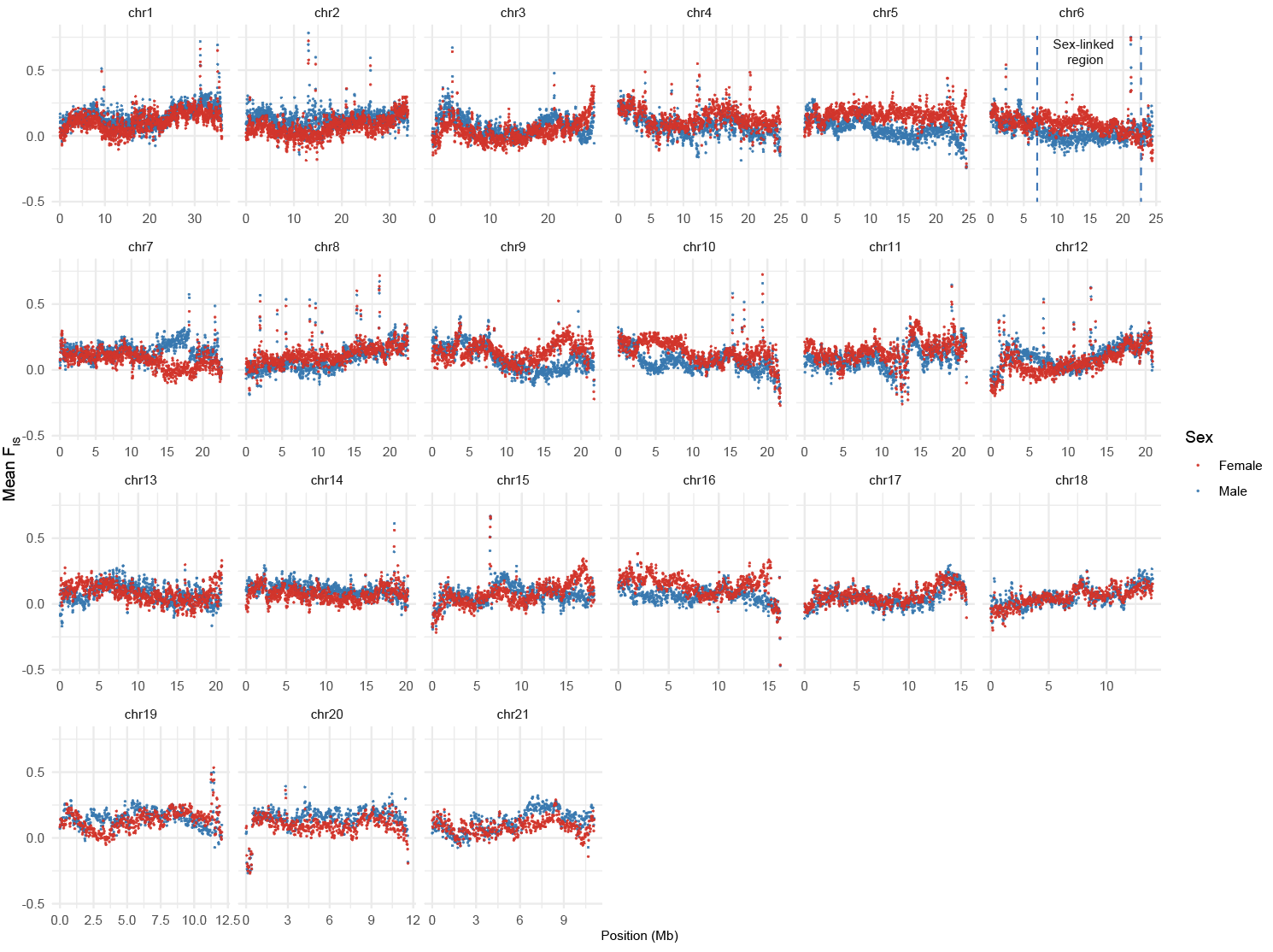


**Supplementary Figure S17. F_IS_ of *H. abdominalis* male and female.**

Mean F_IS_ values in 50Kb windows of male (blue) and female (red) individuals. The sex-linked region on Chr6 (the region between blue dashed lines) has significantly lower F_IS_ values, i.e. higher heterozygosity, in males than in females (p<2.2e-16 by Mann–Whitney U tests), but , though multiple other regions also differed to similar degrees between the two sexes.


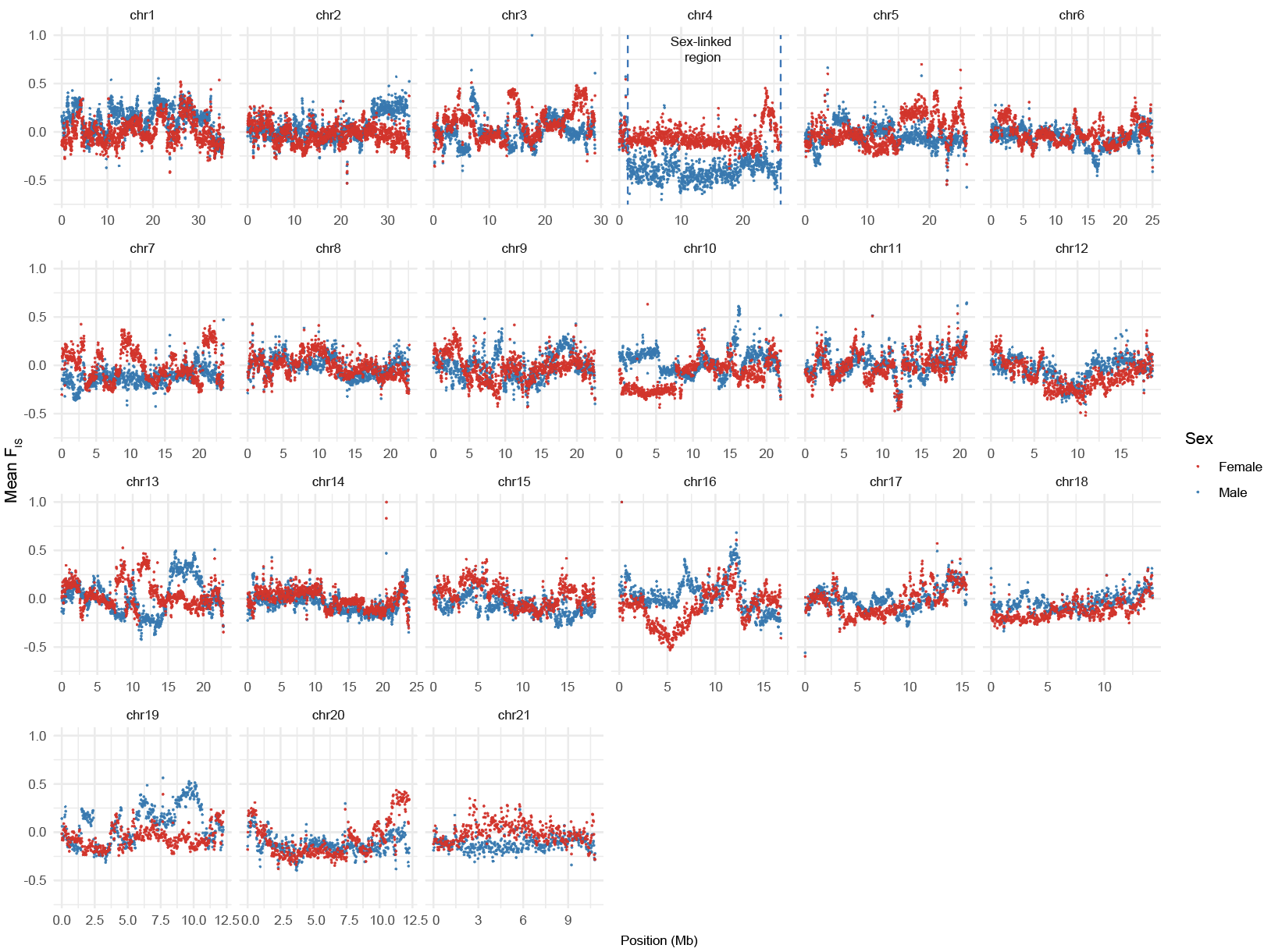


**Supplementary Figure S18. F_IS_ values in *H. erectus* males and females.**

Mean F_IS_ values in 50Kb windows of male (blue) and female (red) individuals. The sex-linked region on Chr4 (between the blue dashed lines) is much more pronounced than in *H. abdominalis*, though again multiple other regions differed between the sexes.


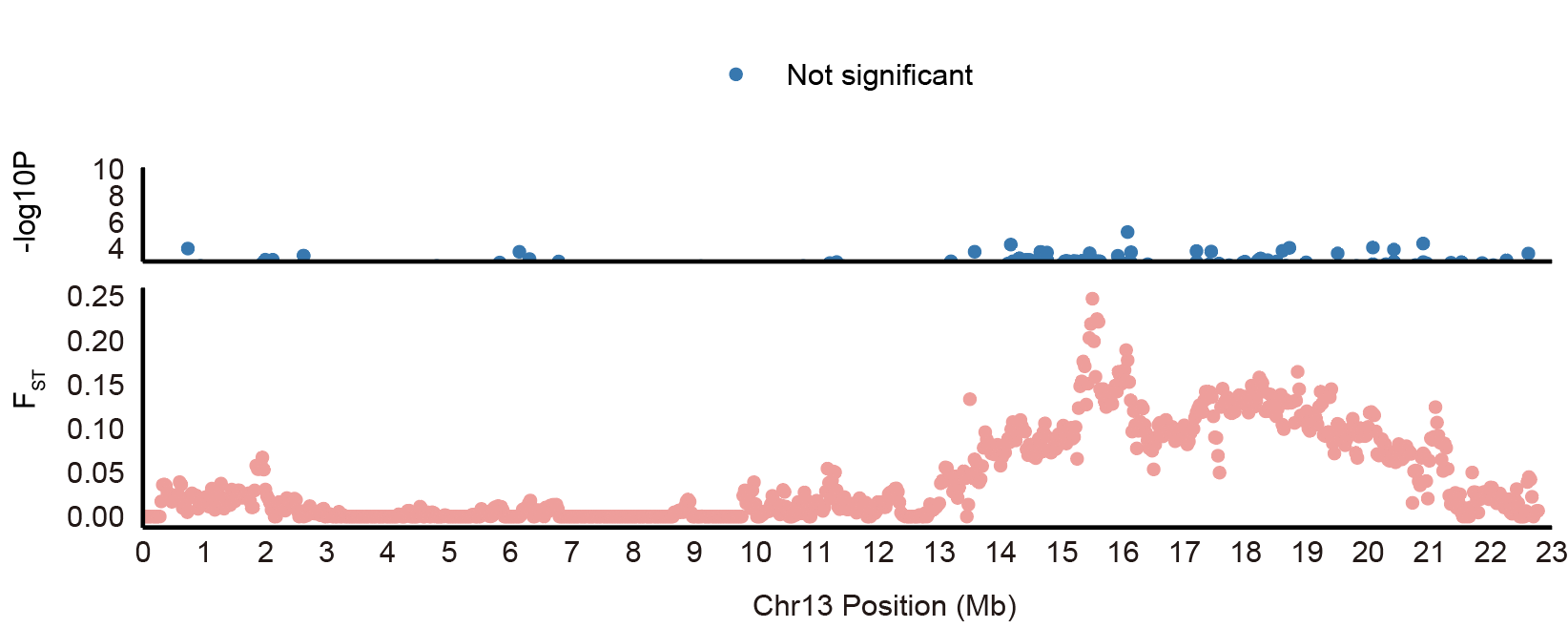


**Supplementary Figure S19. Genetic differentiation between males and females in *H. erectus* Chr13**

SNPs with P <10^-4^ in the GWAS analysis are shown in the upper panel in blue, for compartison with F_ST_ calculated in 50kb-sliding windows (lower panel). The *H. erectus* Chr13 shows an increase in F_ST_ values, but no biallelic SNPs were identified as significantly associated with sex by our GWAS analysis.


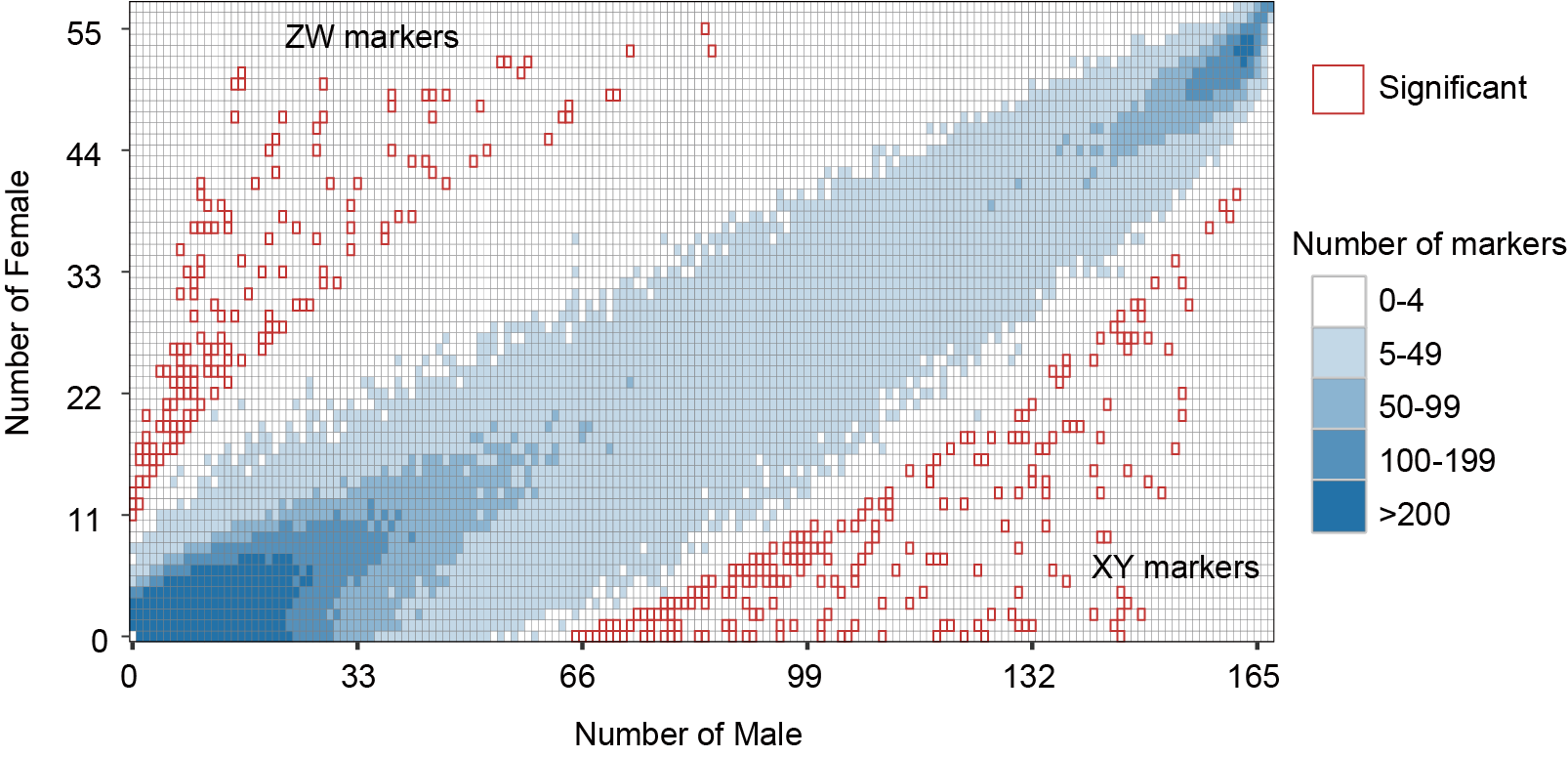


**Supplementary Figure S20. Distribution of RADSex markers in males (horizontal axis) and females (vertical axis).**

Color (blue) intensity for a tile indicates the number of markers present in the corresponding number of males and females. Tiles with significant associations with phenotypic sex (Chi- squared test, p < 0.05 after Bonferroni correction) are indicated with red borders. Significant markers at the bottom right-hand side support an XX/XY sex determination system and ones in the upper left support ZZ/ZW sex determination.

**
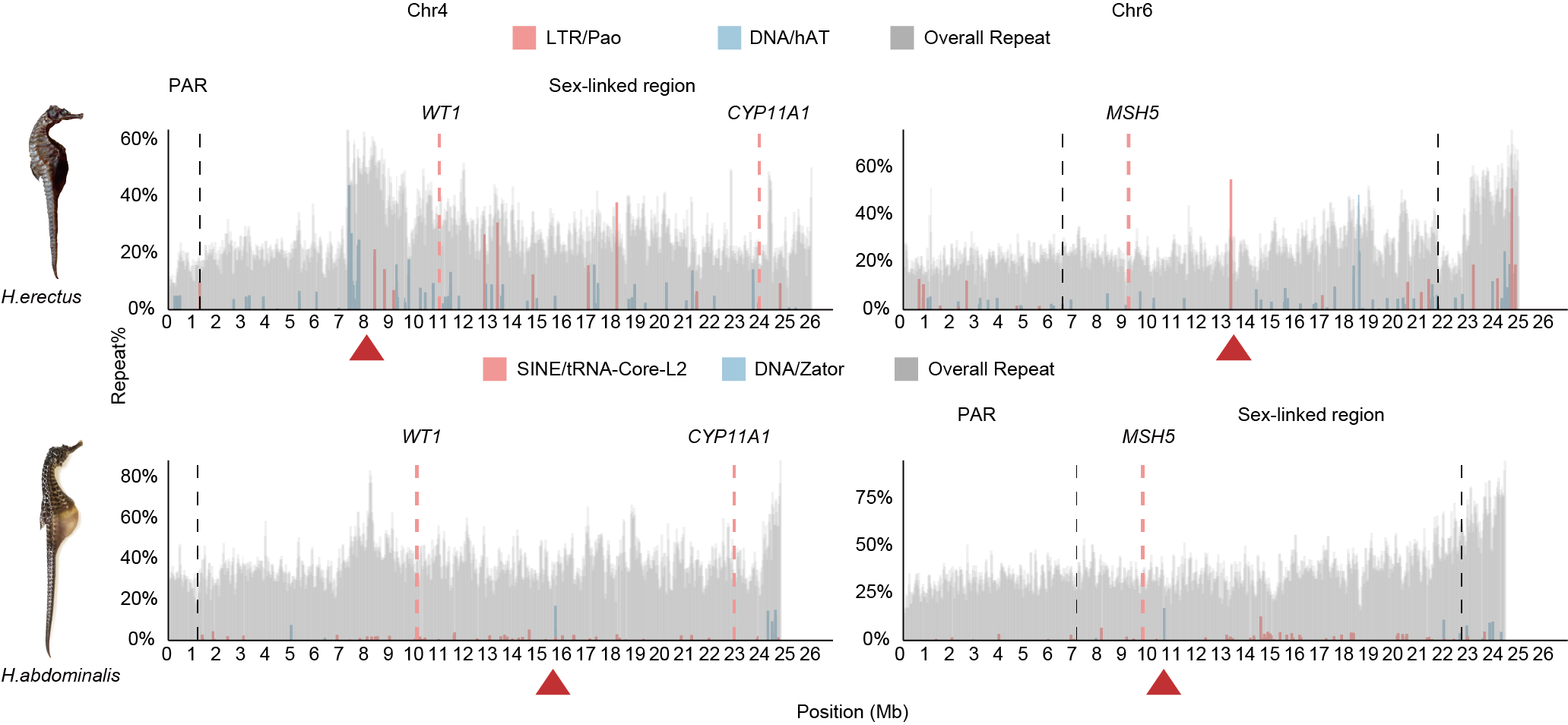
**

**Supplementary Figure S21. Distribution of candidate centromeric repeats in sex-linked region of *H. abdominalis* and *H. erectus*.**

Percentage of candidate centromeric repeat in *H. abdominalis* and *H. erectus* was calculated in each 5kb-sliding window. We found *H. abdominalis* candidate centromeric repeat DNA/Zator located at 11Mb region of Chr6 and both *H. erectus* candidate centromeric repeats DNA/hAT and LTR/Pao were located within sex-linked region of *H. erectus*. And candidate SDG *WT1* and *MSH5* are located near centromeric repeats (labelled by red triangles).


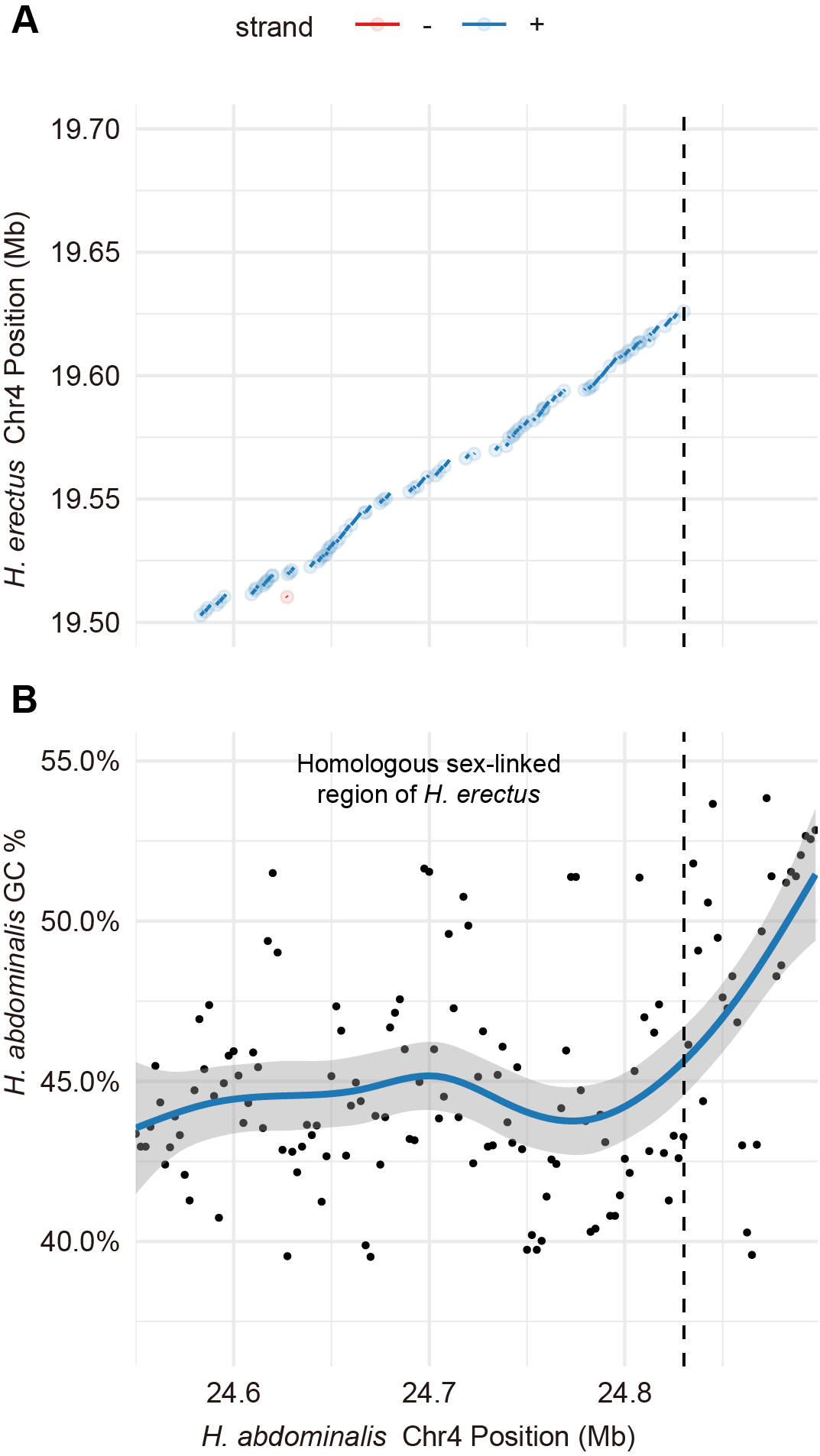


**Supplementary Figure S22. Another possible *H. erectus* PAR homologous to the high-GC region of *H. abdominalis* at the chromosome end**

**(A)** Blue segments represent alignment to the positive strand of *H. erectus*. Red segments represent alignment to the negative strand of *H. erectus.* Dashed line labels the boundary of *H. erectus* chromosome end. **(B)** GC percentage of *H. abdominalis* Chr4 was calculated in each 5kb-sliding window. Loess regression is used to fit a smooth curve for points in scatterplot. On chromosome end of *H. abdominalis* Chr4 there's a high-GC region (At the right side of the dashed line in **B**) which has no homologous region in assembled *H. erectus* genome. We therefore infer that there is another possibly unassembled *H. erectus* PAR homologous to the high-GC region of *H. abdominalis* at the chromosome end.


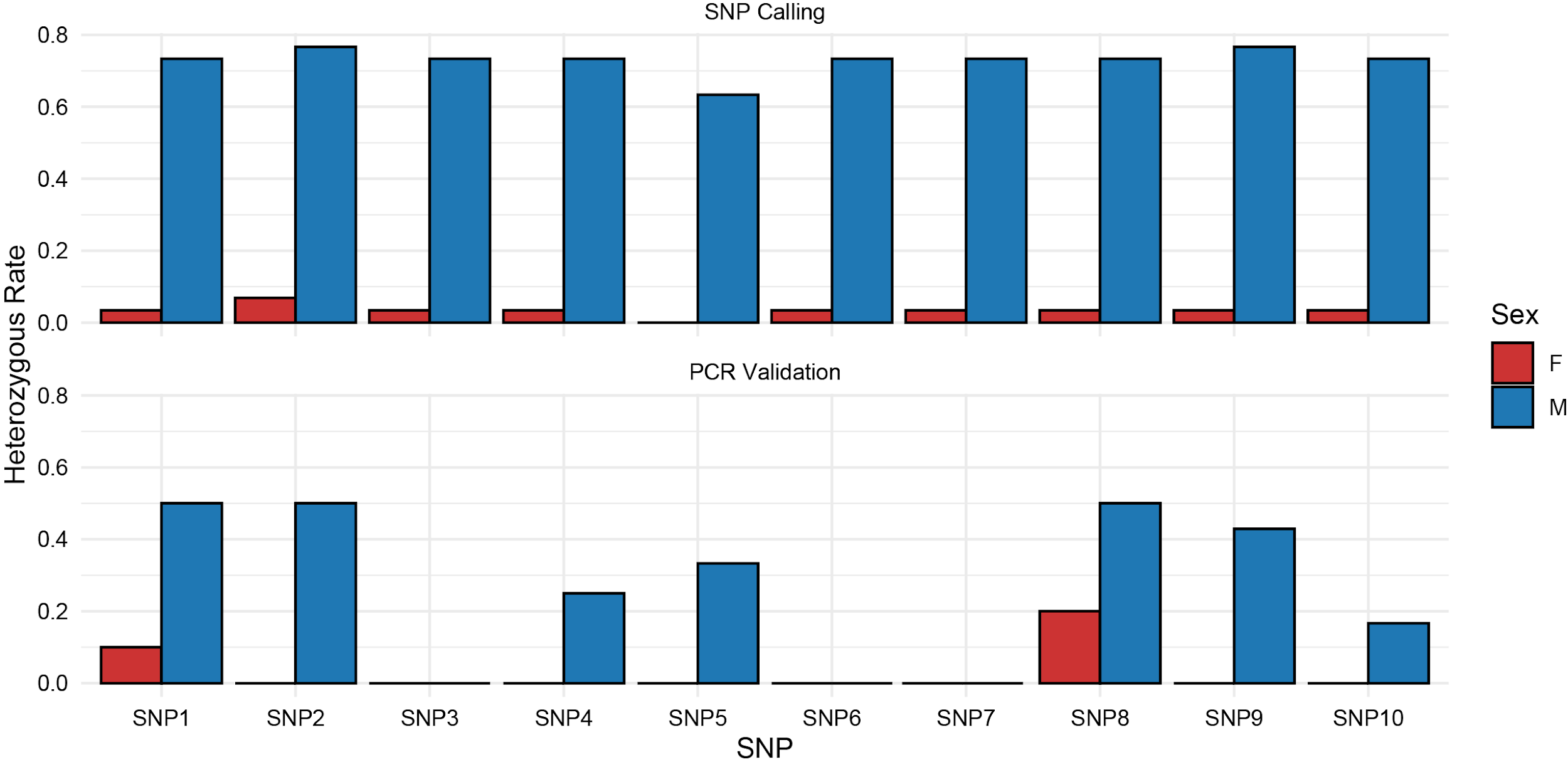


**Supplementary Figure S23. PCR validation of 10 randomly picked sex-associated SNP sites of *H. abdominalis*.**

Validation of ten randomly picked SNPs associated with sex in *H. abdominalis*. The top part shows the evidence for partial associations of the SNPs with sex in our GWAS analyses of sample of 30 *H. abdominalis* individuals of each sex. The y axes of both plots show the frequencies of heterozygotes, illustrating the higher frequencies in males. The bottom part shows the results of PCR genotyping in independent samples consist of 10 male and 10 female individuals. Only seven of the SNPs males had a higher frequency of heterozygotes than females.


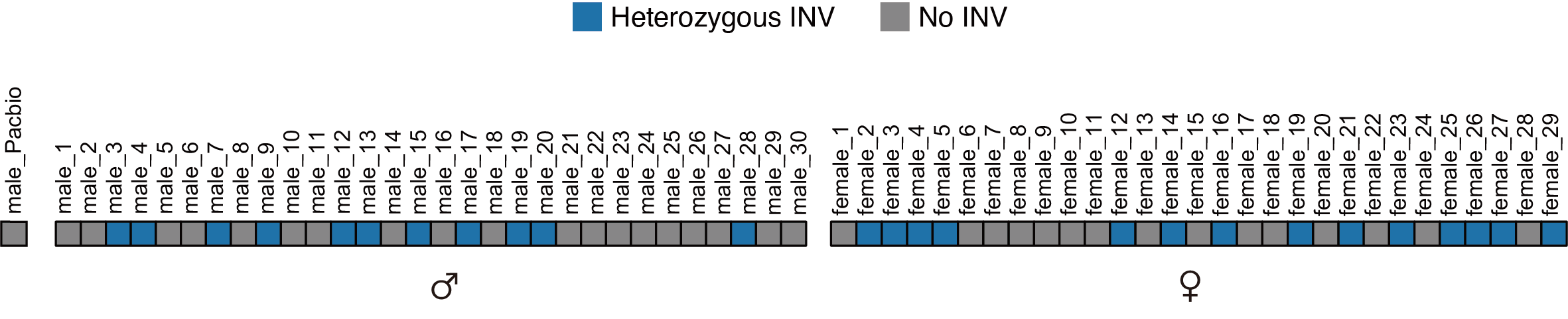


**Supplementary Figure S24. Male and female individuals with reads supporting an inversion near the PAR boundaries of *H. abdominalis*.**

We searched for reads supporting inversions in the genome regions near both PAR boundaries (1.5Mb upstream and downstream of each PAR boundary) and their flanking regions of *H. abdominalis.* In our Illumina (short-read) resequencing reads some individuals of both sexes have at least 2 read pairs supporting an inversion near both PAR boundaries. No sex-specific inversion was detected. PacBio reads from a single male individual found no reads supporting inversions in the regions examined.


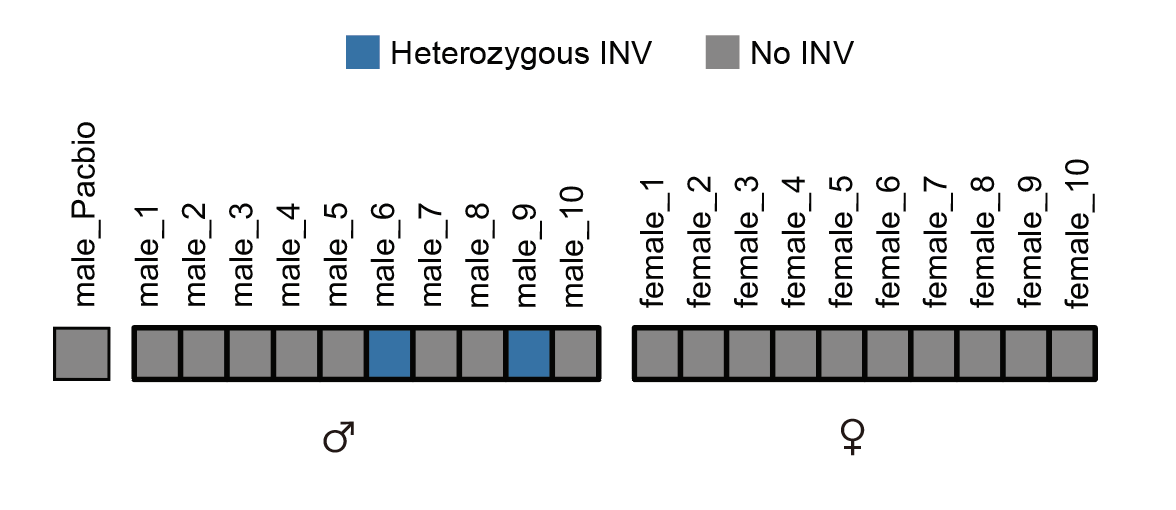


**Supplementary Figure S25. Male and female individuals with reads supporting an inversion near the PAR boundaries of *H. erectus*.**

Our searches for reads supporting an inversion in the *H. erectus* genome region near the PAR boundary (1.5Mb upstream and downstream of the PAR boundary), including the flanking regions*.* Our Illumina (short-read) sequencing reads included only two male individuals with at least 2 read pairs supporting the presence of an inversion, and PacBio reads from a single male Found none. We conclude that the region does not contain any inversion.

**
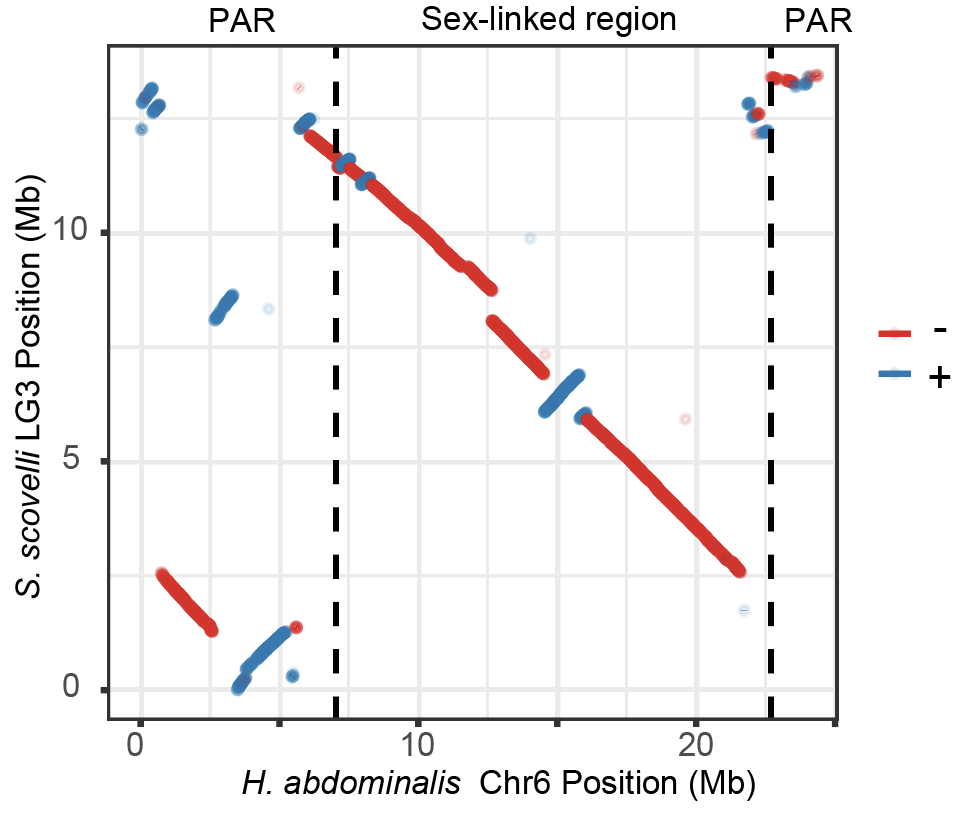
**

**Supplementary Figure S26. Genome alignment between *H. abdominalis* Chr6 and *S.scovelli* LG3**

Blue and red segments, respectively, represent alignment to the positive and negative strands of *S. scovelli*. Alignment between the *H. abdominalis* Chr6 assembly, from a single male individual, and the homologous LG3 of pipefish did not reveal inversions at the PAR boundary.


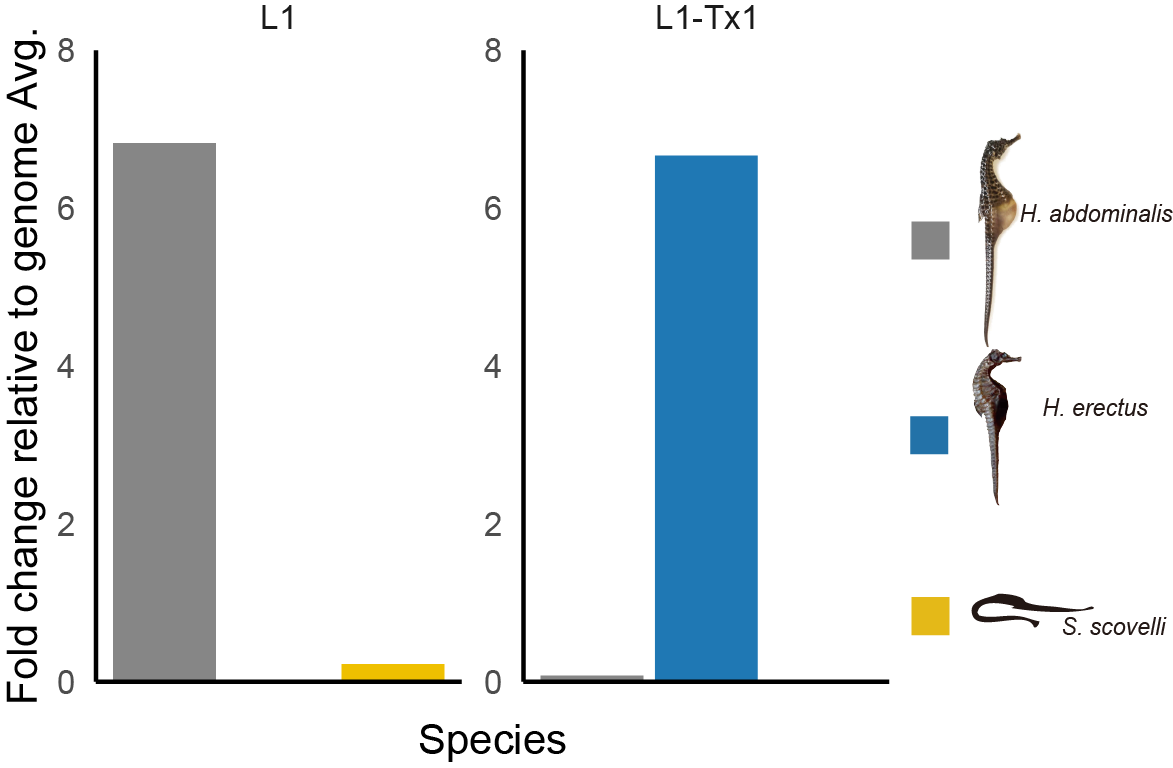


**Supplementary Figure S27. Fold change relative to genome average of sex-linked region boundary-enriched TE subfamilies in other seahorse and pipefish species.**

sex-linked region boundary-enriched TE subfamilies LINE/L1 (6.82-fold enrichment relative to genome average) in *H. abdominalis*, and LINE/L1-Tx1 (6.67-fold enrichment) in *H. erectus* were specifically enriched at their respective boundaries. And these specific TE subfamilies were not found to be enriched at the homologous sequences of the PAR/sex-linked region boundaries in the other seahorse and pipefish species.


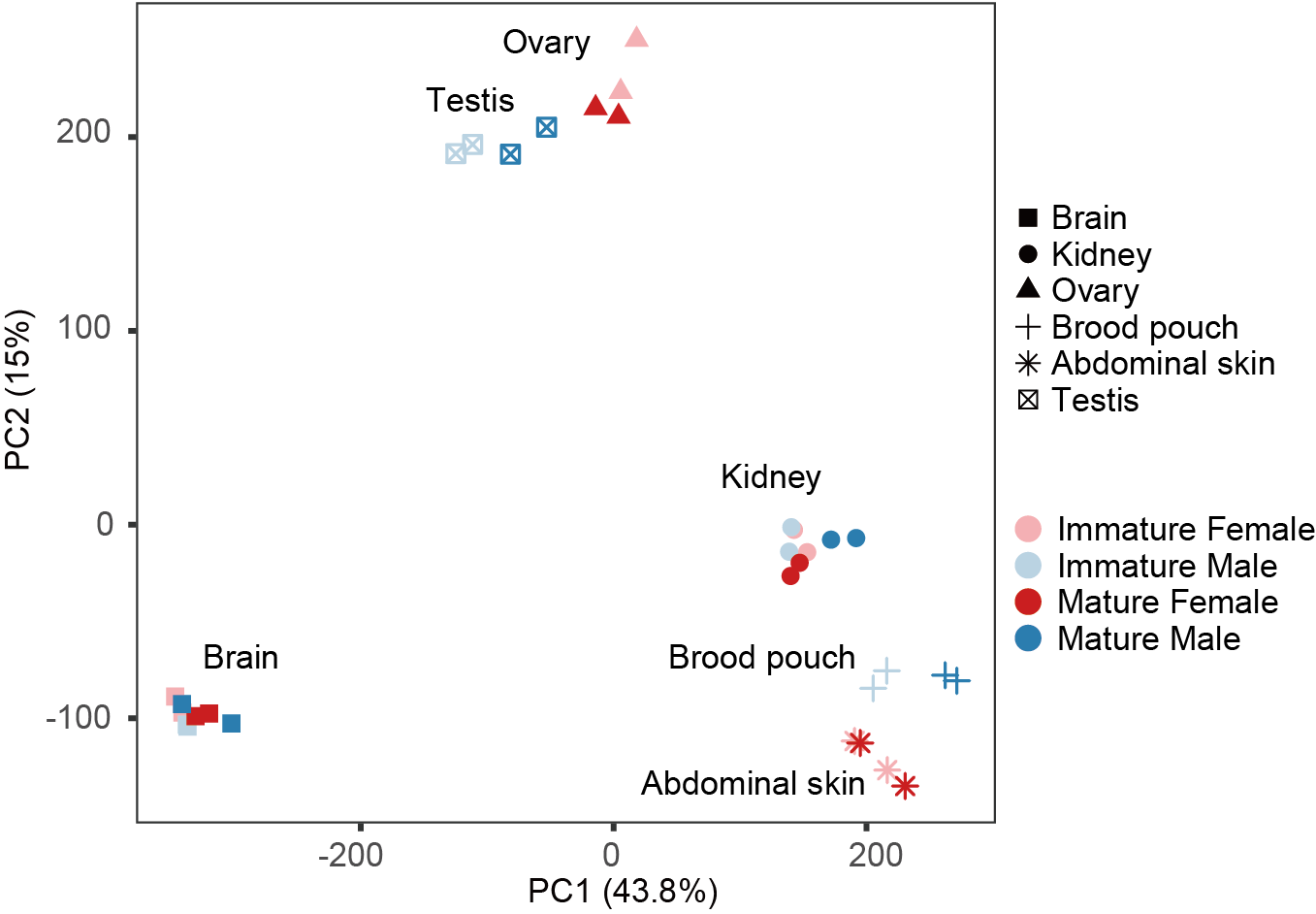


**Supplementary Figure S28. Principal component analysis of collected RNA-seq samples of *H. abdominalis*.**

We collected tissues of immature (3 months) and mature (5 months) stages from brain, kidney, testis, ovary, male brood pouch and female abdominal skin of *H. abdominalis*. For each tissue, we prepared two biological replicates. Read counts were normalized using the TPM (Transcripts Per Kilobase Million) method. Samples are clustered by tissues.


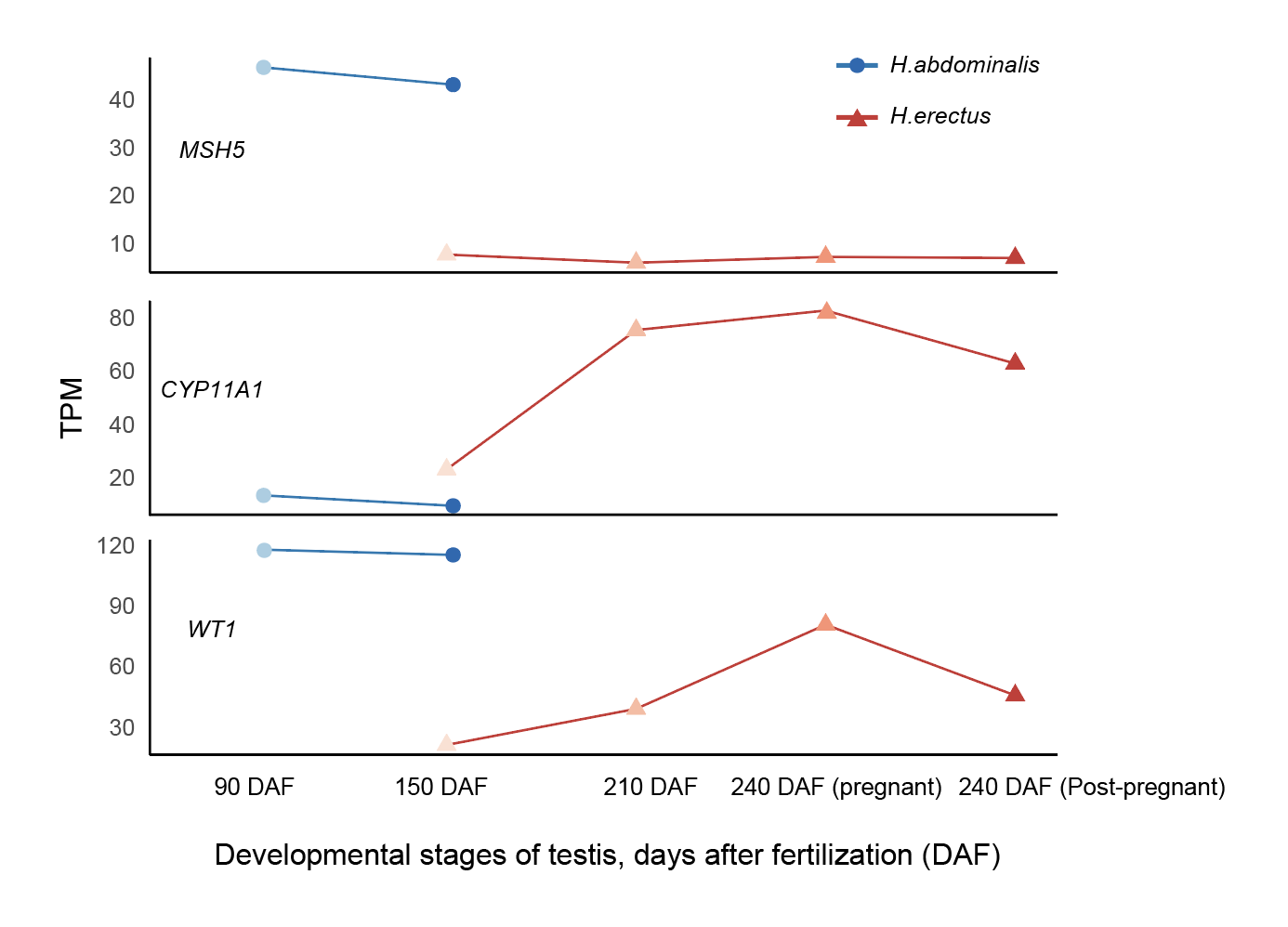


**Supplementary Figure S29. Candidate sex-determining genes' expression across different developmental stages of testis.**

Testis expression of candidate SDGs in *H. abdominalis* and *H. erectus* were presented with blue dots (for *H. abdominalis*) and red triangles (for *H. erectus*). Deeper colors represent later developmental stages. The X-axis indicates different time points. In *H. erectus*, from left to right are testis samples from 150 days after fertilization (DAF), 210 DAF, 240 DAF (pregnant) and 240 DAF (post-pregnant). In *H. abdominalis*, from 90 DAF to 150 DAF.


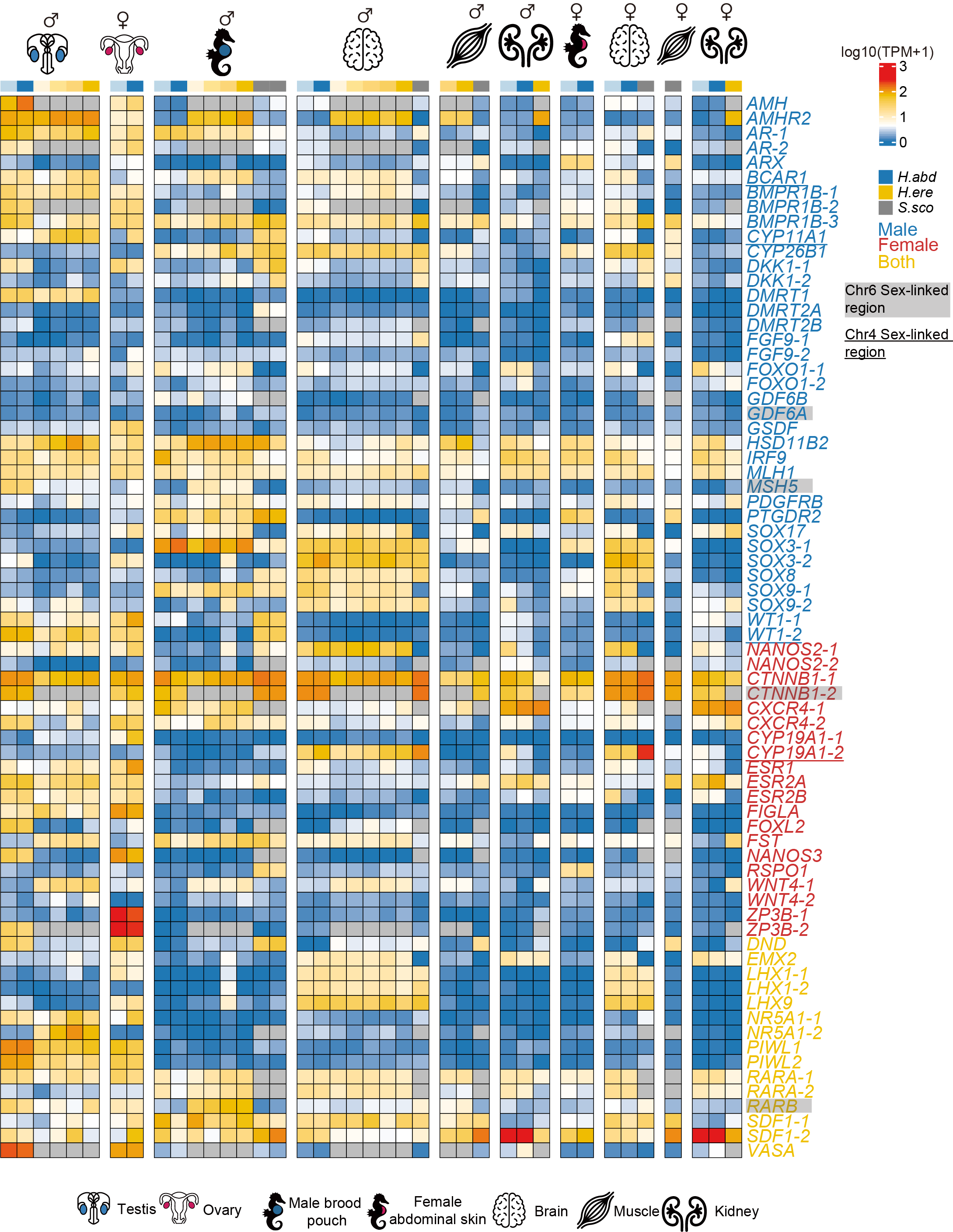


**Supplementary Figure S30. Expression pattern of genes involved in Teleost gonad development in gonads and somatic tissues of *H. abdominalis*, *H. erectus* and *S. scovelli***

Heatmap shows expression patterns of genes involved in Teleost gonad development (Chen, et al. 2018; Guiguen, et al. 2018; Shen and Wang 2018; Taboada, et al. 2018) in different tissues of *H. abdominalis* (blue) and *H. erectus* (yellow) and *S. scovelli* (gray). Deeper colors in the first row represent later developmental stages. Gray boxes represent missing orthologous genes. Genes involved in Teleost gonad development were listed and labelled by different colors. (Blue, red and yellow represent genes involved in Teleost gonad development in male, female and both sexes respectively). -1 and -2 were indicated if there are multiple copies identified across the *H. abdominalis* genome. Shaded genes are on sex-linked region of *H. abdominalis* Chr6 and underlined genes are on sex-linked region of *H. erectus* Chr4.


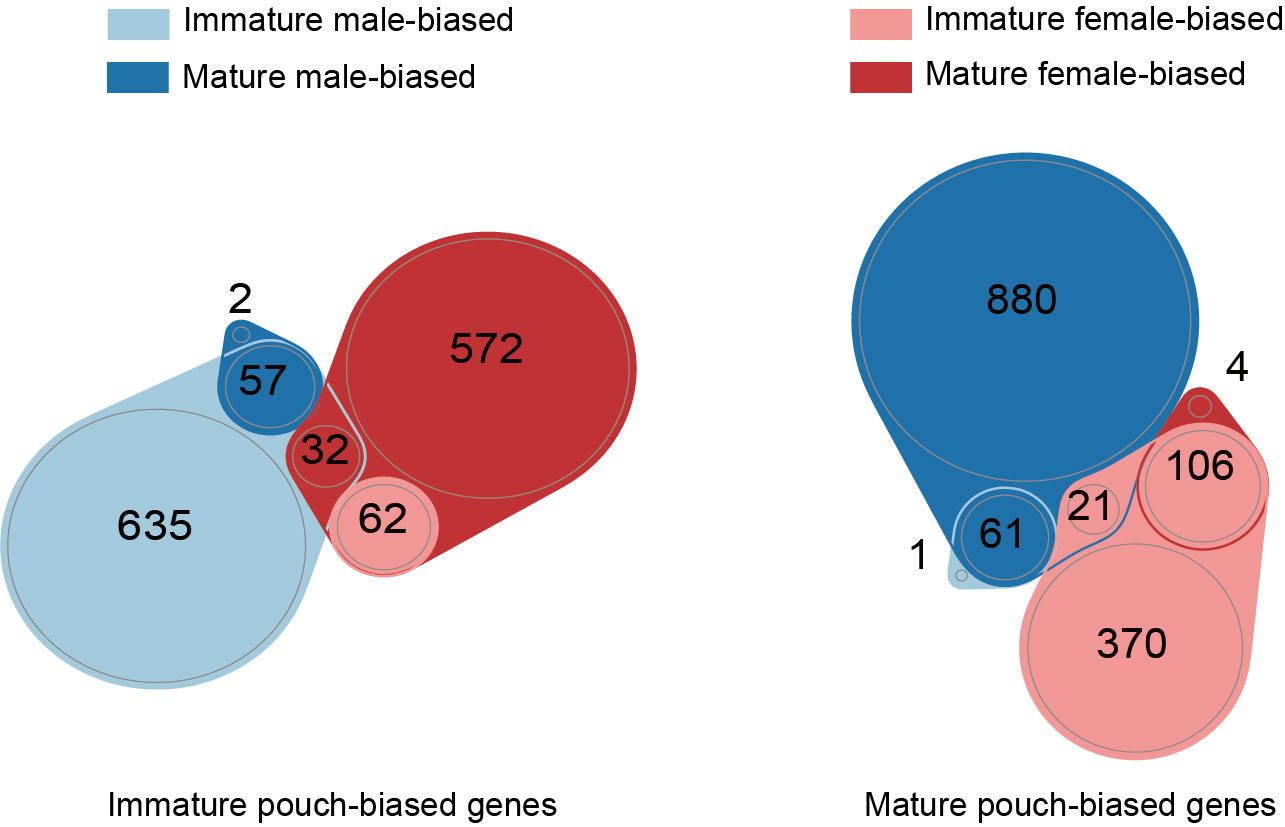


**Supplementary Figure S31. Sex-biasness pattern of pouch developmental genes in *H. abdominalis*.**

Gene expression level in immature pouch and mature pouch were compared with each other. Genes significantly up-regulated in either stage was selected to investigate the sex-biasness pattern. Genes that had a biased transcription level in one certain stage of male brood pouch, were also significantly (P<2.2e-16, Fisher’s exact test) enriched for those biasedly transcribed in the pouch vs. female epidermal tissues of the same stage.


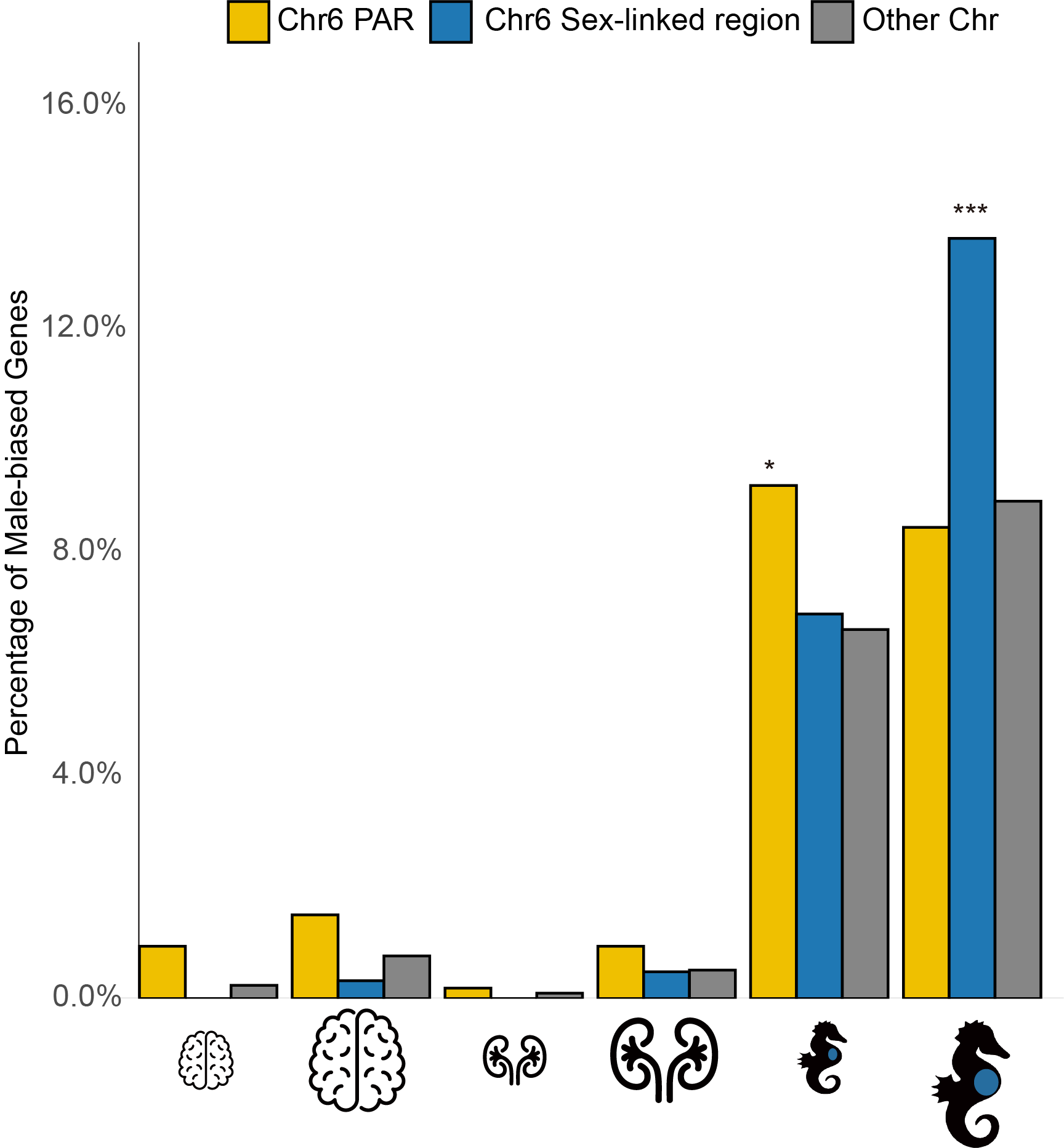


**Supplementary Figure S32. Male-biased genes of *H. abdominalis* take different proportions in different tissues.**

Mature pouch tissue has the highest proportion of male-biased genes, which were defined by comparing the transcriptomes of male pouch vs. female epidermal tissues. Such male-biased genes account for 9.0% of all *H. abdominalis* genes, and there are 6.8% of genes that are male-biased in immature pouch. In other somatic tissues, this number decreases dramatically. Only 0.24%/0.77% of genes in the immature/mature brain and 0.1%/0.52% of genes in the immature/mature kidney are male-biased. Mature pouch-biased genes are also significantly enriched in *H. abdominalis* sex-linked region (P<0.01(**), P<0.001(***), Fisher’s exact test).


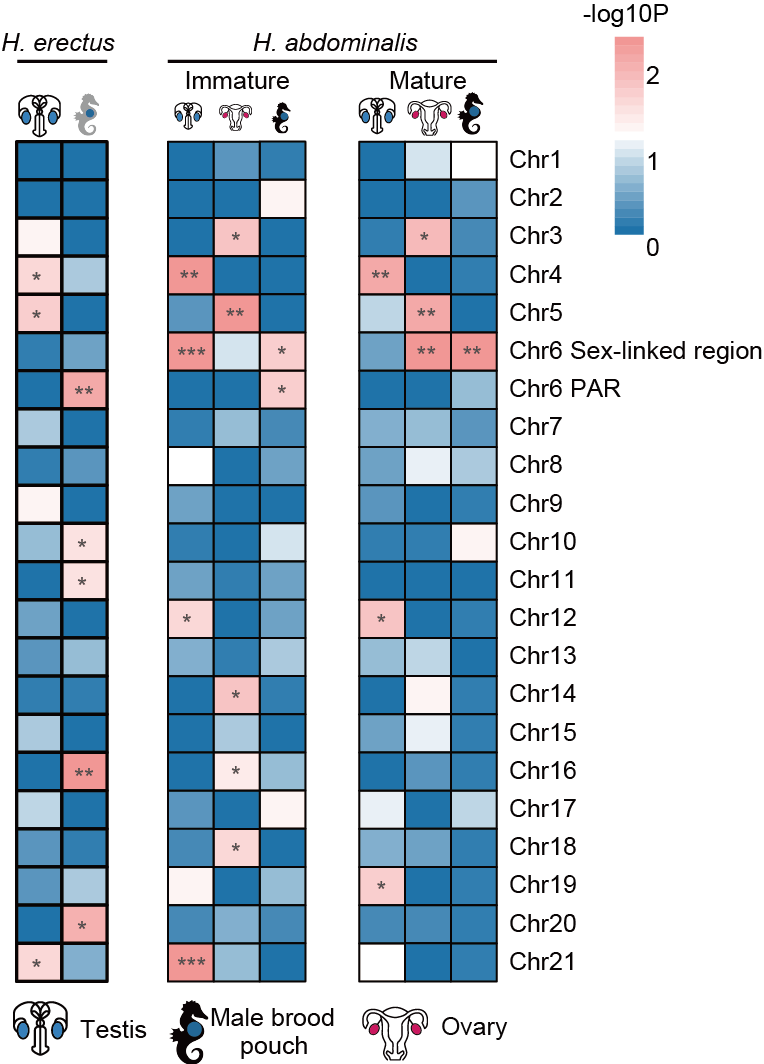


**Supplementary Figure S33. Enrichment of tissue-biased genes on different chromosomes based on TPM fold change.**

Tissue-biased genes were defined if their TPM values were at least two-fold greater than mean TPM of other tissues of the same stage. Heatmap shows the scaled log10 P-values of Fisher’s tests for certain chromosome’s enrichment pattern. Chromosomes significantly enriched for tissue- or male-pouch-biased genes were labelled with asterisks (Fisher’s exact test, *P*<0.05(*), *P*<0.01(**), *P*<0.001(***)).


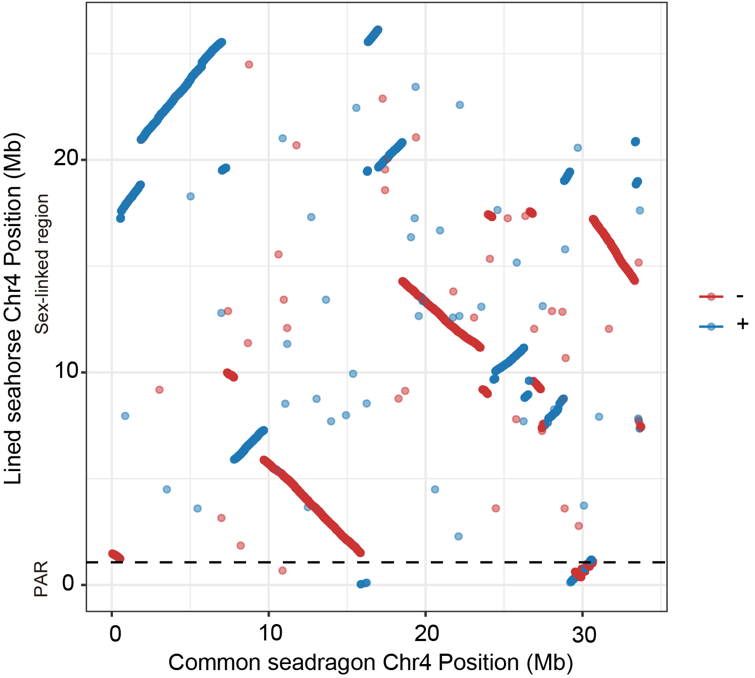


**Supplementary Figure S34. Genome alignment between Common seadragon Chr4 and Lined seahorse Chr4**

Dashed line labelled boundary between PAR and sex-linked region of Chr4 of Lined seahorse. Blue segments represent alignment to the positive strand of Nile tilapia. Red segments represent alignment to the negative strand of Nile tilapia. Chr4 of Lined seahorse is homologous to Chr4 of Common seadragon.


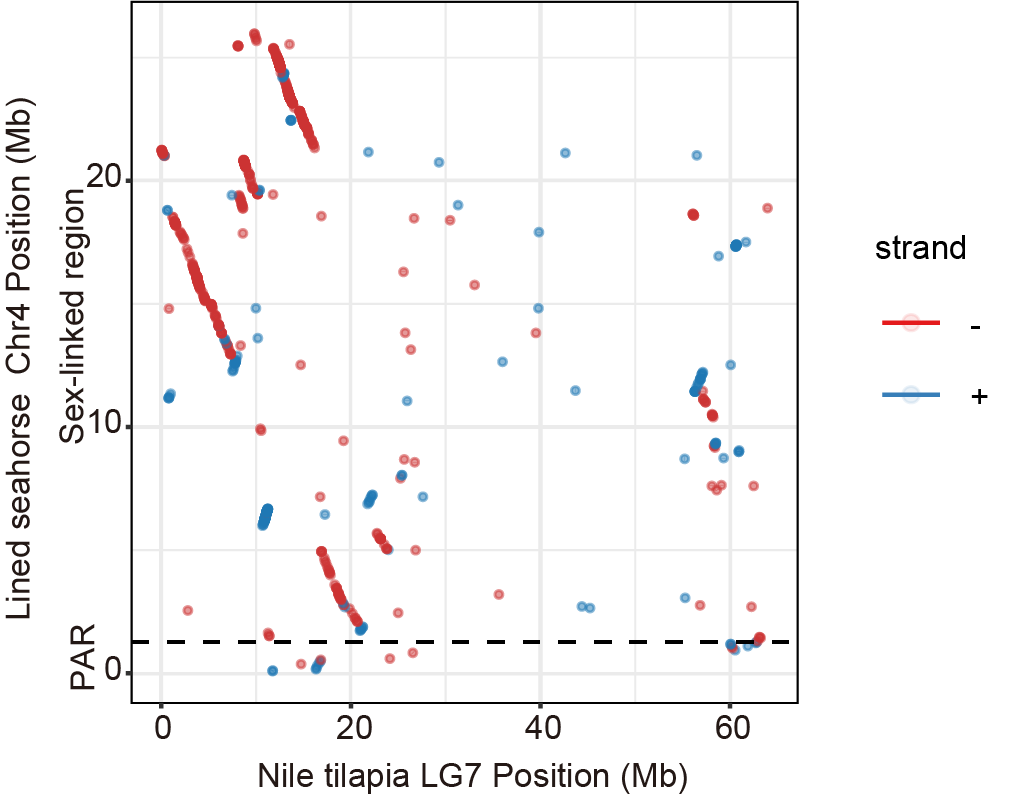


**Supplementary Figure S35. Genome alignment between Nile tilapia LG7 and Lined seahorse Chr4**

Dashed line labelled boundary between PAR and sex-linked region of Chr4 of Lined seahorse. Blue segments represent alignment to the positive strand of Nile tilapia. Red segments represent alignment to the negative strand of Nile tilapia. Chr4 of Lined seahorse is homologous to 21Mb of the LG7 of Nile tilapia.

**References**

Chen S-L, Zhou Q, Shao C-W. 2018. Genomic and Epigenetic Aspects of Sex Determination in Half-Smooth Tongue Sole. In. Sex Control in Aquaculture. p. 525-545.

Guiguen Y, Fostier A, Herpin A. 2018. Sex Determination and Differentiation in Fish. In. Sex Control in Aquaculture. p. 35-63.

Killick R, Eckley I. 2014. changepoint:an R package for changepoint analysis. J. Stat. Softw. 58:19.

Shen Z-G, Wang H-P. 2018. Environmental Sex Determination and Sex Differentiation in Teleosts – How Sex Is Established. In. Sex Control in Aquaculture. p. 85-115.

Taboada X, Robledo D, Bouza C, Piferrer F, Viñas AM, Martínez P. 2018. Reproduction and Sex Control in Turbot. In. Sex Control in Aquaculture. p. 565-582.
